# Keratin Additive for Cellular Adhesion in Transcutaneous Prosthetics

**DOI:** 10.1155/term/4337554

**Published:** 2025-12-30

**Authors:** A. L. Cagle, E. L. Szulc, J. Flaggert, Y. Arias, A. Nikhar, D. Tadio, J. A. Durant, I. L. Gitajn, K. R. Hixon

**Affiliations:** ^1^ Thayer School of Engineering, Dartmouth College, Hanover, New Hampshire, 03755, USA, dartmouth.edu; ^2^ Geisel School of Medicine, Dartmouth College, Hanover, New Hampshire, 03755, USA, dartmouth.edu

**Keywords:** cryogel, electrospinning, implants, keratin, percutaneous, tissue engineering, transcutaneous

## Abstract

The dermal barrier is widely considered the body’s first line of defense against most foreign bodies, protecting it from both moisture loss and bacterial invasion. However, when the skin is ruptured for long‐term medical interventions (e.g., transcutaneous prosthetics), it is difficult to restore and maintain this protective barrier. Although there are no direct, biological examples of true transcutaneous features in the human body, similar phenomena can be observed in phalangeal nails. This study aims to investigate keratin, the primary component of fingernails, in its hydrolyzed form as an additive to induce cell adhesion in two representative scaffold types. Electrospun fibers and chitosan–gelatin cryogels—two well‐characterized scaffolds used in dermal tissue engineering—were selected for this study as a fibrous and macroporous foundation. Both electrospun fibers and cryogels were fabricated with a range of keratin additive concentrations (0, 1, 3, 5, 7, and 10 wt/wt% and wt/v% for electrospun fibers and cryogels, respectively) and tested for surface properties, mechanical strength, biocompatibility, and material behavior. Overall, it was determined that hydrolyzed keratin had a positive effect on cell adhesion and proliferation but that high quantities of the keratin resulted in adverse effects on the scaffold properties. With dermal applications in mind, this study found that 5 and 7 wt/wt% keratin electrospun fibers possessed required cell counts, surface energies, tensile strength, and contact angle, all with consistent reproducibility. For the cryogels, 3 and 5 wt/v% keratin had the best combined performance, maintained structural integrity through swelling and porosity, and displayed minimal loss in compressive strength. Therefore, hydrolyzed keratin represents a promising additive for bothelectrospun fibers and cryogels in tissue engineering applications.

## 1. Introduction

### 1.1. Transcutaneous Prosthetics

Transcutaneous implants and long‐term transdermal devices have become indispensable in modern medicine, offering innovative solutions for long‐term conditions. Technologies such as osseointegration, colostomy bags, and permanent intravenous (IV) ports have revolutionized treatment approaches and improved patient outcomes [1–3]. These devices not only restore function but also significantly improve quality of life and patient care. Osseointegration, which allows direct skeletal attachment of prosthetic limbs [1, 4], greatly enhances stability and functionality of the devices. Colostomy bags provide crucial support for individuals with intestinal or bowel complications, enabling them to maintain hygiene and a sense of normalcy in their daily lives [5]. Permanent IV ports serve as reliable access points for long‐term IV therapies, reducing the discomfort and inconvenience associated with frequent needle insertions [6]. These devices represent examples of transcutaneous medical technology advancements that have positively impacted patient care and quality of life.

Despite their clinical benefits, all transcutaneous implants share a fundamental biological challenge: establishing a stable and protective interface at the site where the device breaches the skin and underlying soft tissue. This junction often fails to form an effective seal, leaving the percutaneous opening prone to irritation, epithelial downgrowth, and microbial invasion. Osseointegration prosthetics exemplify these risks, as their direct skeletal anchoring leaves the skin–implant interface particularly vulnerable to infection, marsupialization (epithelial downgrowth), and irritation as outside contaminants can find easy passage through the unsecured barrier [7, 8]. Infection poses a distinct threat to transcutaneous implants, particularly osseointegrated prosthetics, as it is difficult to treat once infection occurs. Additionally, the subsequent inflammatory response can result in demineralization around the implant, loosening, and overall failure at the tissue (e.g., bone) interface which is especially relevant for osseointegrated prosthetics [9–11].

### 1.2. The Dermal Barrier

The skin, composed of the epidermis and dermis, serves as the body’s largest organ [12] and offers protection against environmental threats such as ultraviolet (UV) radiation, pathogens, and dehydration [12, 13]. Its intricate structure involves layers with specific functions: (i) The epidermis, rich in keratinocytes, contains accessories such as hair follicles and sweat glands, and (ii) the dermis, composed of fibrous connective tissue, provides strength and elasticity [14]. Wound healing, a crucial process for organism survival, involves complex mechanisms such as clotting, inflammation, cellular proliferation, and extracellular matrix (ECM) remodeling [14]. Commonly, wound repair of any large magnitude results in fibrotic tissue formation or scarring, which increases the difficulty of dermal reattachment to a foreign device [15].

### 1.3. Tissue Engineering and Keratin

Tissue engineering offers a promising approach to biomedical applications, providing a matrix for tissue repair, replacement, and/or regeneration. A wide range of scaffold fabrication techniques have been developed to target the formation of this tissue matrix while encouraging tissue integration. Although previous attempts to secure the dermal barrier around transcutaneous prosthetics or permanent stomas have lacked substantial promise, tissue engineering approaches present a potential solution [7, 16–18]. Previous studies have attempted to induce dermal adhesion directly to metal implants using surfacing and surface treatments with limited success [7, 16]. Furthermore, additional studies have explored biomimetic materials to replicate the natural dermal adhesion to transcutaneous appendages found in many species of antlered mammals [18–21]. This study expands on the biomimetic approach but aims to imitate an interface that is prevalent in humans. Although the human body does not contain any known mechanism to support the passage of bone tissue through the dermis, it does support the adhesion of dermal cells to keratinous appendages (i.e., fingernails) [22]. Keratins, a family of ubiquitous, fibrous proteins [23–25], play a crucial role in cell adhesion through interactions with supramolecular complexes [26]. Extracted keratins have already been used as a coating to promote cell attachment to scaffolding materials [27]. With the known phenomenon of keratinous fingernails protruding through the dermal barrier, the incorporation of keratin as a scaffold additive holds significant promise for enhancing dermal adhesion and ingrowth for transcutaneous prosthetics.

Despite being a promising component for inducing cell adhesion, keratin cannot support cellular activity by itself; biomaterial scaffolding is required for structural support. In particular, macroporous gels and electrospun fibers (ESFs) are commonly used in dermal tissue engineering [28, 29]. Although skin standardly exhibits accelerated healing as compared to other tissues in the body, the presence of transcutaneous elements in the healing site necessitates extended scaffolding support and a slower degradation timeline. Therefore, natural polymer chitosan–gelatin cryogels (CGs) could be used, as they exhibit an in vivo degradation timeline of approximately 1 year [30]. Synthetic polycaprolactone (PCL) ESFs also represent a useful option, and together, these are among the most commonly used materials in tissue‐engineered scaffolding.

### 1.4. Electrospinning and ESFs

Electrospinning has emerged as a versatile technique with numerous benefits for dermal engineering applications [30]. The fibrous structure produced through electrospinning is highly advantageous as it can closely mimic the natural ECM of tissues, providing a biomimetic environment for cell growth and tissue regeneration [31]. These ESFs offer a high surface area‐to‐volume ratio, promoting cell adhesion, proliferation, and differentiation [32]. Additionally, the properties of ESFs are tunable, allowing for control over parameters such as fiber diameter, alignment, porosity, and mechanical strength [33]. This tunability enables customization to match specific tissue requirements, making electrospinning a promising approach in the field of dermal and soft tissue engineering.

Despite these advantages, electrospinning natural polymers alone often results in fibers with poor durability and mechanical fragility, limiting their translational potential. To overcome these limitations, blending natural and synthetic polymers has been explored as a strategy to combine the bioactivity of natural polymers with the mechanical robustness of synthetics. In this work, we therefore focused on keratin–PCL blends as a representative natural–synthetic system that leverages the strengths of both material classes. PCL has emerged as a valuable electrospinning material for dermal and soft tissue engineering due to its customizable degradation rate, mechanical properties, simple shaping capabilities, and potential for controlled drug delivery [34, 35]. Its intrinsic biocompatibility and biodegradability have established PCL as a preferred choice across tissue engineering applications [35]. Moreover, PCL’s role in promoting repair and regeneration in various tissues, namely bone and skin, underscores its versatility [35, 36]. Collectively, the tunable properties of PCL‐based systems make them particularly well‐suited for supporting tissue reconstruction and enabling efficient drug delivery applications, further enhancing their utility in biomedical settings [34].

### 1.5. Chitosan–Gelatin CGs

Chitosan–gelatin CGs have also emerged as a promising avenue for soft tissue engineering, offering a range of benefits for regeneration. Characterized by their macroporous structure formed through crosslinking at subzero temperatures, CGs provide interconnected pores that facilitate cell distribution within the scaffold [37]. Natural polymers such as chitosan and gelatin have been extensively studied for their biodegradability, biocompatibility, renewability, and low cost, making them ideal candidates for CG fabrication [37–39]. Chitosan, a natural polysaccharide derived from chitin, possesses biocompatibility, biodegradability, antimicrobial properties, and the ability to promote cell adhesion and proliferation [40–42]. Gelatin, a denatured form of collagen, offers similar advantages and cell‐binding motifs (i.e., the arginine–glycine–aspartic acid [RGD] sequence) to enhance cell attachment [43]. These natural polymers not only imitate elements of the ECM but also interact favorably with various cell types, making them valuable components in tissue engineering strategies [39, 44]. The combination of chitosan and gelatin in composite scaffolds or CGs has shown great potential in tissue engineering by providing a supportive environment for cell growth, differentiation, and tissue regeneration [37, 38]. In particular, the highly porous and interconnected network of CGs provides ample space for cell hosting, thereby promoting infiltration throughout the scaffold [37]. This unique structure not only supports soft tissue reconstruction but also allows for effective drug delivery applications due to its tunable properties and biocompatibility [38, 45, 46]. Unlike ESFs, CGs composed entirely of natural polymers can achieve sufficient structural integrity and slower degradation profiles, making them well‐suited for applications where mechanical brittleness is less of a concern. Thus, the use of natural polymer‐based CGs complements the natural–synthetic ESF approach, together providing a balanced material strategy for a combined scaffold design.

### 1.6. Combination Construct

This study represents the first phase in developing a combination tissue construct designed to address the weaknesses of transcutaneous implants and long‐term transdermal devices (e.g., osseointegration). The composite scaffold integrates (i) ESFs, serving as an artificial basement membrane to support fibroblastic adhesion at the dermal interface, and (ii) a chitosan–gelatin CG, providing structure and reinforcement to the fiber network.

Our objective is to evaluate cell adhesion to both PCL ESFs and chitosan–gelatin CGs when supplemented with keratin. By incorporating keratin into these well‐characterized scaffold platforms, we aim to enhance dermal adhesion and tissue ingrowth at the skin–implant interface. This work seeks to generate translational insights into scaffold design for transcutaneous prosthetics, advancing strategies in both tissue engineering and regenerative medicine.

## 2. Materials and Methods

### 2.1. Scaffold Fabrication

#### 2.1.1. Fabricating ESFs

Electrospinning solutions (*N* = 3) were prepared by dissolving 5 wt/v% PCL pellets in tetrafluoroethylene (TFE) overnight with constant stirring until a clear solution was obtained [47]. Varying concentrations of K3030 hydrolyzed keratin (2000 Da; sourced from ovine and porcine hair and hides from Spectrum Chemical [CAS # 69430‐36‐0]; incorporated at 0, 1, 3, 5, 7, and 10 wt/wt%) [48, 49] were then dissolved in deionized (DI) water and incorporated into the PCL/TFE solution. The resulting electrospinning solution (see Table 1) was then gently stirred until homogeneity was achieved [47].

**Table 1:** Electrospun keratin–PCL scaffold composition ratios.

Keratin (%)	PCL (g)	Keratin (g)	DI water (mL)	TFE (mL)
0	1.00	0.00	0.00	10.00
1	1.00	0.01	0.10	9.90
3	1.00	0.03	0.30	9.70
5	1.00	0.05	0.50	9.50
7	1.00	0.07	0.70	9.30
10	1.00	0.10	1.00	9.00

The electrospinning process was conducted by filling a 20 cc syringe with the final 10 mL of solution and carefully removing any bubbles. An 18‐gauge, stainless steel needle was then mounted onto the syringe pump assembly of an electrospinner (Spinbox Advanced Electrospinning). A working distance of 15 cm and a flow rate of 3 mL/hour were maintained for each solution, and each sheet was collected for 2 h. A 10‐cm‐diameter rotating barrel apparatus was used and set to 1600 rpm to align fibers [50]. Positive and negative voltages (0–20 kV, 0‐(‐10) kV) were set to stabilize the Taylor cone and prevent erroneous fiber drift. Atmospheric temperatures varied within room temperature (RT) conditions (∼22°C and ∼43% humidity). Collected samples for subsequent testing were trimmed to remove any thinning outer regions and stored within a desiccator at RT to ensure complete drying and prevent moisture contamination over time.

#### 2.1.2. Fabricating CGs

The chitosan–gelatin solution was prepared by dissolving 8 wt/v% chitosan powder in a 1v/v% acetic acid solution on a mechanical mixer with constant stirring until a homogeneous mixture was achieved [28]. Then, 32 wt/v% gelatin powder was added to the chitosan solution and mixed again [28]. Varying concentrations of keratin (0, 1, 3, 5, 7, and 10 wt/v%) were incorporated into the chitosan–gelatin mixture and stirred until homogeneity was achieved. CG formation was initiated through a combination of a 2.5 v/v% glutaraldehyde solution, decanted thoroughly into the polymer solution. After homogenizing, the resulting combined solution was poured into syringes that had been prechilled at −20°C. Filled syringes were then placed back into the −20°C freezer and allowed to crosslink and freeze for 18 h.

Once formed, CGs were ejected from the syringes while still frozen. CGs were then sectioned and frozen at −80°C for ≥ 1 h before being lyophilized (Labconco FreeZone Benchtop Freeze Dryer) overnight to completely remove all ice crystals and moisture from the macropores. Dried CGs were then stored at RT in a desiccator until experimental use.

### 2.2. Surface Characterization

#### 2.2.1. Scanning Electron Microscopy Pore and Fiber Analysis

All scaffolds were sputter‐coated (Hummer 6.2 Sputter Coater) and subsequently imaged under a scanning electron microscope (SEM; Tescan VEGA3). SEM imaging of scaffold surfaces was performed to confirm porosity and topographical features before cell seeding. The resulting SEM images were analyzed using PoreVision [51], a pore analysis Python program to measure the pore areas of both the CGs and ESFs. Additional measurement of the ESF diameters was performed manually with random sample selection of fibers (*N* = 60) per sample image (*N* = 3) in ImageJ.

#### 2.2.2. ESF Contact Angle

ESFs were evaluated using a goniometer setup [52] to measure contact angle and subsequent surface energy. To accomplish this, fibers were sectioned into ∼0.5 cm × 0.2 cm rectangular strips and suspended between three‐dimensional (3D)‐printed clips to ensure gentle but stable tension while avoiding wrinkles in the fiber mat. Next, a droplet (7 μL) of DI water was dispensed using a vertically suspended micropipette. A camera recorded the dispensing from the tip of the pipette to the surface of the ESFs, and screen capture was used to retrieve images from seconds 2, 5, 10, and 25, after droplet contact with the fiber surface. ImageJ was then used to quantify the angle between droplet edges and scaffold surface on the retrieved images.

#### 2.2.3. CG Swelling Kinetics

CGs were subjected to a 24‐h swell test, involving submersion and subsequent weight measurements of the water‐saturated gels to confirm structural integrity and retention of swelling capabilities. This evaluation method was based off of previous studies [53–55].

### 2.3. Mechanical Testing

#### 2.3.1. ESF Tensile Testing

ESFs underwent tensile testing (Instron 68SC‐2) with clamp attachments. Fibers were loaded until failure to assess maximum tensile strength and elastic modulus, as well as the modulus at 10% strain. To thoroughly understand the mechanical behaviors, we loaded fiber samples both along their fiber alignment direction such that the fibers were (vertically) loaded along their long axes, as well as perpendicular to their alignment direction such that the loading pulled the fibers (laterally) apart.

#### 2.3.2. CG Compression Testing

CGs also underwent hydrated mechanical testing to 90% compression (Instron 68SC‐2) with a 500N max load cell to assess elastic modulus and compression strength. Measurements were taken throughout the compression process, as well as the stress at the final (90%) compression value and the compression moduli at 10% and 50% compression.

### 2.4. Degradation and Leach Testing

#### 2.4.1. Mass Gain/Loss Test

Complete media used for all following tests consisted of high‐glucose Dulbecco’s modified Eagle’s medium (DMEM) with 10% fetal bovine serum (FBS) and 1% penicillin–streptomycin (pen‐strep; 10,000 U/mL source concentration). The degradation of both scaffold types was tested with a 7‐ and 14‐day mass loss evaluation. Briefly, dry scaffolds were first placed in media and weighed. Scaffolds were then allowed to remain in media for 7 and 14 days in a CO_2_ incubator at 37°C, 5% CO_2_. At Day 7, scaffolds in the first group were reweighed, frozen, and lyophilized. SEM imaging was performed to visualize the scaffold surface morphology and detect signs of structural degradation. For the second group, the media were changed on Day 7 and mass measurement, lyophilization, and SEM imaging occurred on Day 14.

#### 2.4.2. Scratch Assay for Identifying Cytotoxic Solutes

Scaffolds were placed in complete media and allowed to rest in a CO_2_ incubator at 37°C, 5% CO_2_ for 72 h. Scaffolds were then removed, and the conditioned media were collected and frozen at −20°C. Adult human dermal fibroblasts (HDFas; ATCC) were cultured in a 24‐well plate with unconditioned complete media to confluency. Once confluent, all wells were scratched with a 100‐μL pipette tip in a cross formation. The initial image for the *t* = 0 h time point was positioned such that the vertical scratch was centered and the horizontal scratch was just out of frame. This ensured consistency of location with the paired images. After the scratch, the thawed, conditioned media were gently pipetted into each well and cells were allowed to proliferate for 24 h in the CO_2_ incubator. Scratch assay images were taken on an EVOS M5000 Microscope at 10× zoom. The scratch closure was assessed using the wound‐healing size tool ImageJ plugin developed by Suarez‐Arnedo et al. using the wound‐healing size tool and the wound‐healing size manual tool [56]. Wound closure was then calculated using the formula: [(Initial wound area ‐ current wound area)/initial wound area] × 100%.

### 2.5. *In Vitro* Culture

#### 2.5.1. Scaffold Cell Culturing

Electrospun scaffolds were placed into a 48‐well plate. Fibers were disinfected via 1‐h UV sterilization on either side within the well plate. CGs were also placed into a 48‐well plate and disinfected via two 30‐min ethanol rinses followed by three 10‐minute phosphate‐buffered saline (PBS) rinses. Both groups of disinfected scaffolds were then seeded with 50,000 HDFas in ∼200 µL of high‐glucose DMEM modified with 10% FBS and 1% pen‐strep as previously described. All well plates were placed in a CO_2_ incubator, and cells were allowed to adhere for 1 hour following seeding. After the adherence period, additional media were gently dispensed into the well to reach a total of 400 µL of high‐glucose DMEM per scaffold. Scaffolds were cultured within the incubators, and analyses were performed on Days 1, 3, and 5 after seeding. Scaffolds undergoing imaging were removed from the media at the endpoints and submerged in 1‐2 mL of paraformaldehyde for 20 min at RT. Following the fixation period, scaffolds were transferred into PBS for storage at 4°C. All remaining scaffolds underwent cellular proliferation quantification on Days 1, 3, and 5.

#### 2.5.2. Cell Study Evaluation

Following cell seeding, a tetrazolium compound [3‐(4,5‐dimethylthiazol‐2‐yl)‐5‐(3‐carboxymethoxyphenyl)‐2‐(4‐sulfophenyl)‐2H‐tetrazolium], inner salt (MTS)/phenazine methosulfate (PMS) cell proliferation assay was performed (CellTiter 96 Aqueous Non‐Radioactive Cell Proliferation Assay, Promega). A standard curve was established using cultured HDFas (*N* = 3) in a sterile 48‐well plate with 400 µL of media. Plated cells were allowed an hour to adhere to the standard curve well plate. In the experimental scaffold wells, the old medium was replaced with fresh medium before proceeding with the proliferation assay. Following this, 80 μL (20% wt/v) MTS/PMS was added to each well and incubated in a 5% CO_2_ incubator to protect from light. After one hour, 120 μL of resuspended assay/media from both the standard curve and the scaffolds was collected via pipette and plated in a 96‐well plate. The plate was then read on a SpectraMax Paradigm Multi‐Mode Detection Platform plate reader at 490nm, and the optical values were recorded. The standard curve was used to establish a second‐order regression curve, and cell counts were assigned based on the resulting equation.

Fixed ESF and CG scaffolds were imaged on a confocal microscope (Andor Spinning Disk) to examine z‐stacked cell migration. Preparation of scaffolds postfixing involved sample blocking with a BSA and Tween [20] buffer before staining with an multispecies reactivity antivinculin–FITC mouse monoclonal antibody in a humidified chamber for 2 h. After primary staining, scaffolds were rinsed in PBS and stained again with a secondary goat antirabbit IgG (H + L) tagged with Alexa Fluor 488 for 2 h in a humidified environment protected from visible light. Samples were then rinsed again in PBS and stained with 4′,6‐diamidino‐2‐phenylindole (DAPI) for 20–40 min in a dark, humid environment. All scaffolds were rinsed in PBS to remove excess stain. Confocal images were collected with a 60× oil lens (*N* = 3 per scaffold group). Z‐stack files were collected and processed using Fiji on ImageJ.

## 3. Results

### 3.1. Surface Characterization

#### 3.1.1. ESF Surface Characterization

ESF pores were measured via SEM (Figures 1(a) and 1(b)) coupled with PoreVision [47] analysis software. Contact angle was also measured and averaged to evaluate the wetting behavior of the scaffolds (Figure 1(c)). SEM imaging and PoreVision [47] surface analyses of ESF scaffolds (Figures 1(d) and 1(e)) reveal a general trend of decreasing pore area and perimeter, respectively, with increasing keratin concentration via the 1‐way Brown–Forsythe and Welch ANOVA (*N* = 3 images from three different sample scaffolds; *p* < 0.05). Fiber diameters were also confirmed to decrease with increasing keratin concentration (Figure 1(f)). Confirmation of this trend was achieved using the 1‐way Brown–Forsythe and Welch ANOVA with 60 randomized measurements (*N* = 3 independent samples; *p* < 0.05).

Figure 1Representative SEMs of PCL ESFs with increasing keratin concentration (0–10 wt/wt%) at (a) 1000× and (b) 7000×. (c) Representative images of the water droplets measured for contact angle at *t* = 10 s showing a visible decrease in contact angle after 5 wt/wt%. (d) ESF pore area analysis showing a significant decrease in pore area in all groups except for 3%. (e) ESF pore analysis (*N* = 3) showing significant decreases in pore perimeter at 5% and 10% keratin. (f) ESF fiber diameter analysis (*N* = 3) showing a significant decrease from the 0% group for the 7 and 10 wt/wt% groups. (g) Contact angle analysis (*N* = 3) showing a slight increase in contact angle with low keratin concentrations followed by a rapid and inconsistent drop in contact angle in the 7 and 10 wt/wt% groups.

**Figure:**
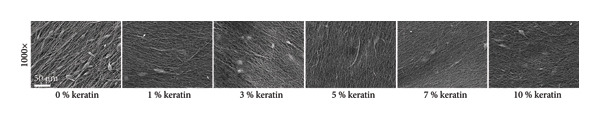
(a)

**Figure:**
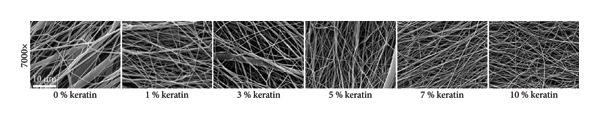
(b)

**Figure:**
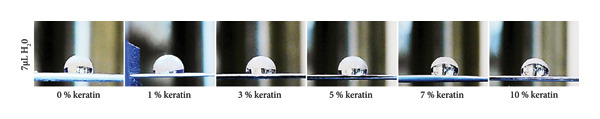
(c)

**Figure:**
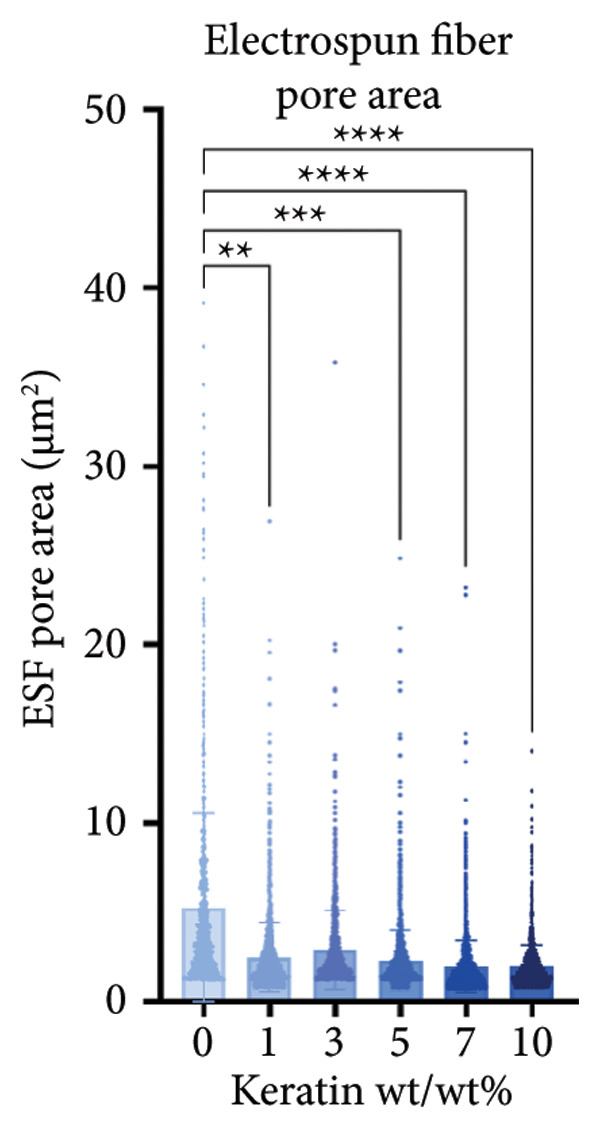
(d)

**Figure:**
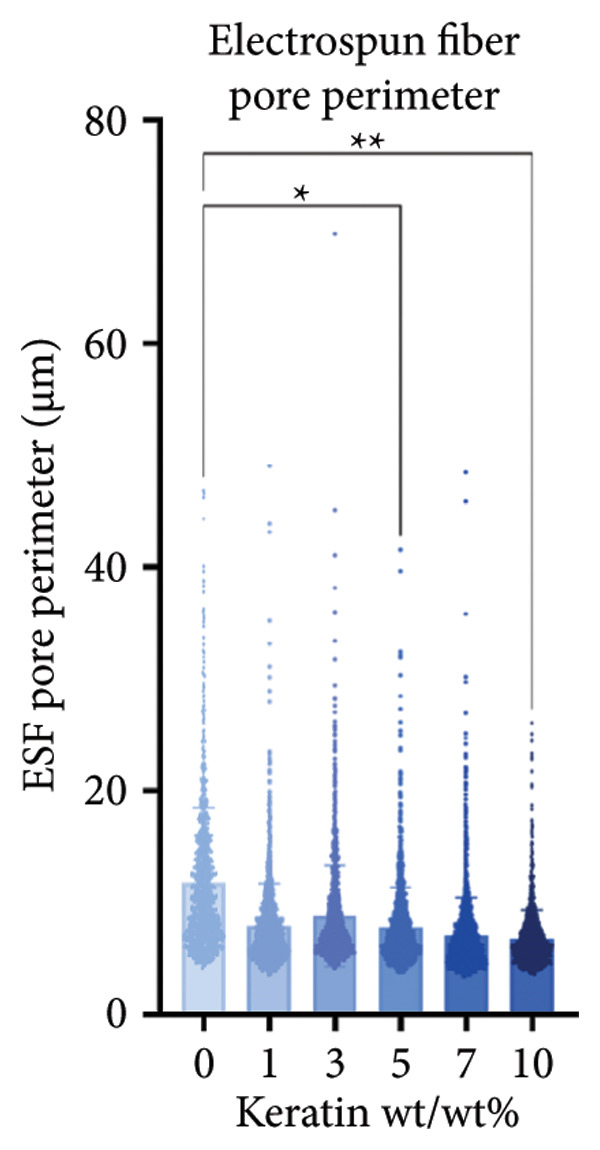
(e)

**Figure:**
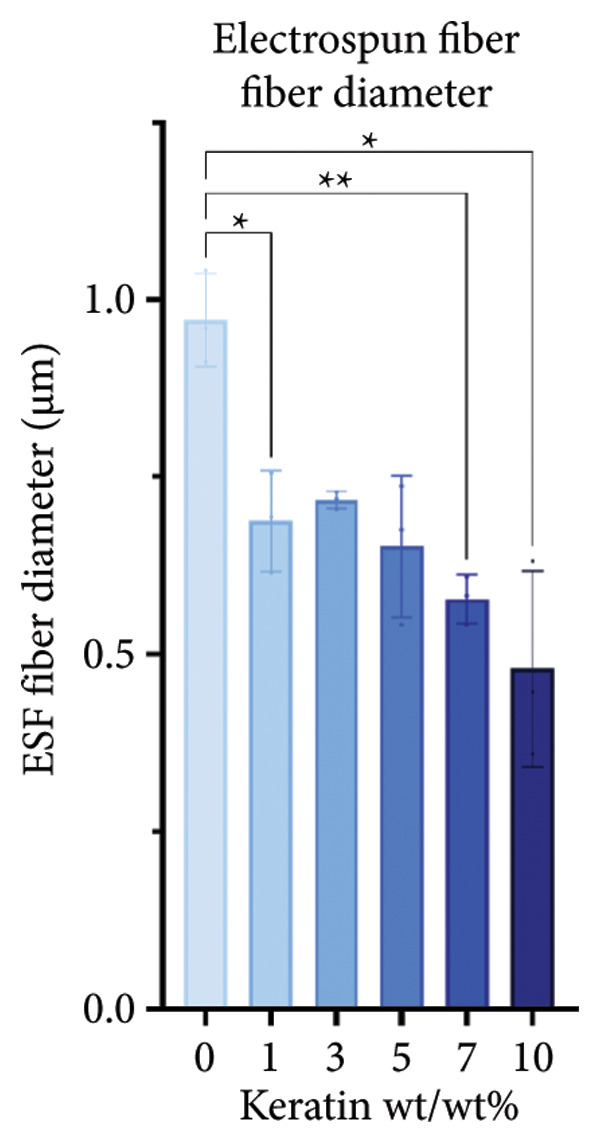
(f)

**Figure:**
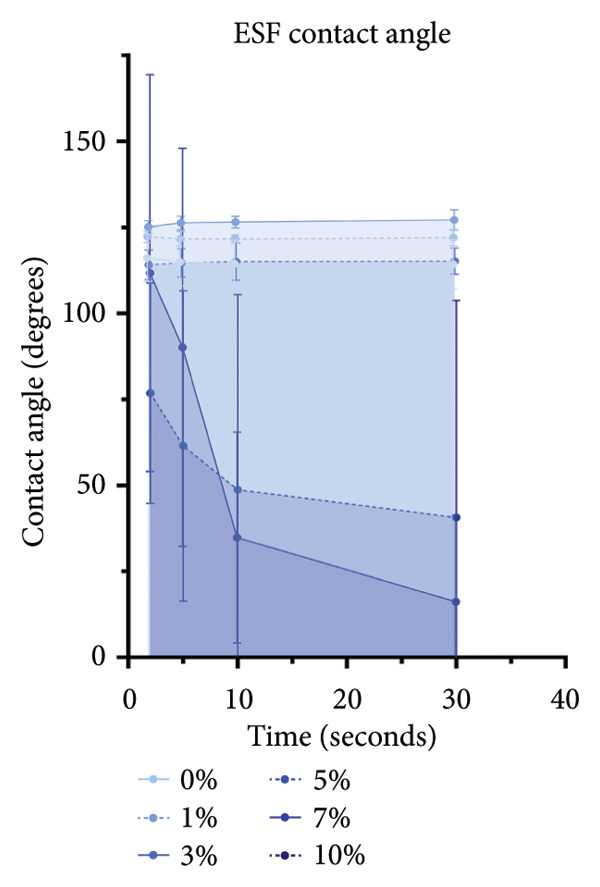
(g)

Scaffold wettability evaluations using the 3D‐printed goniometer setup demonstrate significant changes in contact angle between the different keratin fibers (Figure 1(g)). Expanded significance can be found in Supporting Figure 1. These results were confirmed using 2‐way ANOVA with Tukey’s correction for multiple comparisons with a single pooled variance. Both left and right contact angles were included for each sample (*N* = 6). For samples with keratin concentrations of 7 and 10 wt/wt%, wettability was significantly increased and droplets were rapidly absorbed into the fibers. In contrast, for scaffolds with 0–5 wt/wt%, the droplets consistently remained intact for the full 30 s measured. This indicates that increasing the concentration of keratin in the fibers significantly increases, around the 7% wt/wt% mark, the surface energy and hydrophilicity of the scaffold surface, which could majorly affect surface adhesion. This visible trend was confirmed by a full‐effects model 2‐way ANOVA with Tukey’s correction for multiple comparisons compared within time points and across keratin percentages.

#### 3.1.2. CG Surface Characterization

CG pores were evaluated using SEM images and PoreVision software [47] of the scaffold surface (Figures 2(a) and 2(b)). Images were also taken of the gels before and after hydration to demonstrate the effect of water on the gel volume and characteristics (Figure 2(c)). Analysis demonstrated significant changes in pore area (Figure 2(d)) but no changes in pore perimeter (Figure 2(e)) with increasing keratin. This indicates that the formation of CG networks is more resilient to increasing quantities of the keratin additive than the pore networks in the ESFs. However, the CG appearance did visually darken when soaked in water with increasing keratin; the 7 wt/v% scaffolds were darker in appearance when hydrated than the 0 wt/v% CGs despite little visual difference when dry (Figure 2(c)). The CG structure also changed drastically at the 10 wt/v% mark. Furthermore, these scaffolds, when hydrated, were extremely fragile and more hydrogel‐like in texture as compared to the spongy 0–7 wt/v% CG scaffolds.

Figure 2Representative SEMs of CGs with increasing keratin concentration (0–10 wt/v%) at (a) 100× and (b) 500×. (c) Representative images of CGs (left) before and (right) after being submerged in water for 24 h, showing a noticeable decrease in structural integrity in 10% keratin. (d) CG pore analysis showing no significant decreases in pore perimeter with increasing keratin (*N* = 3). (e) CG pore area analysis showing a significant increase in pore area for 3%, 5%, and 7% groups (*N* = 3). (f) Swell test for CGs (*N* = 6) showing statistically diminished swelling capacity in groups with keratin percentages of 3%, 5%, and 10% and a visual trend of decreasing swell capability with increasing keratin.

**Figure:**
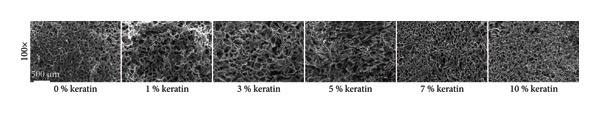
(a)

**Figure:**
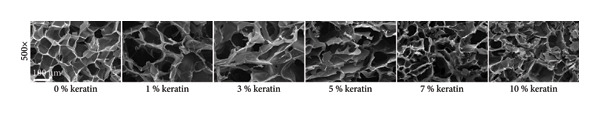
(b)

**Figure:**
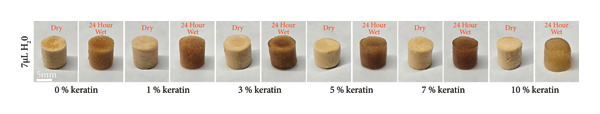
(c)

**Figure:**
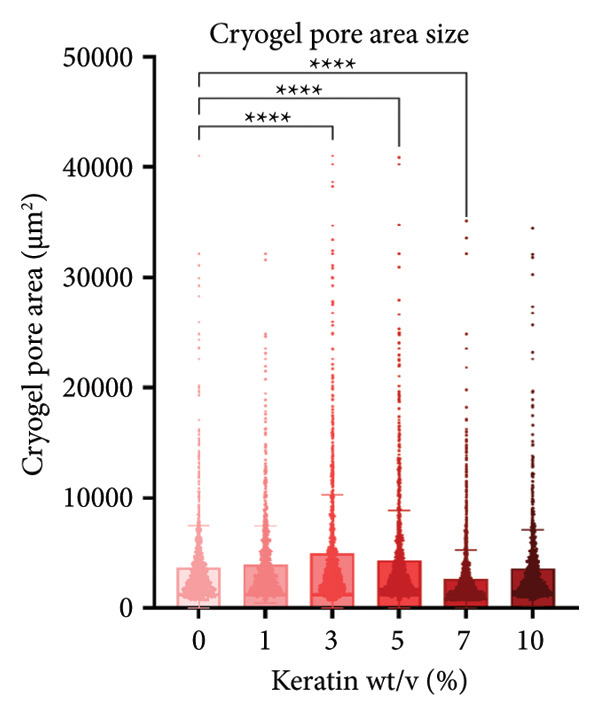
(d)

**Figure:**
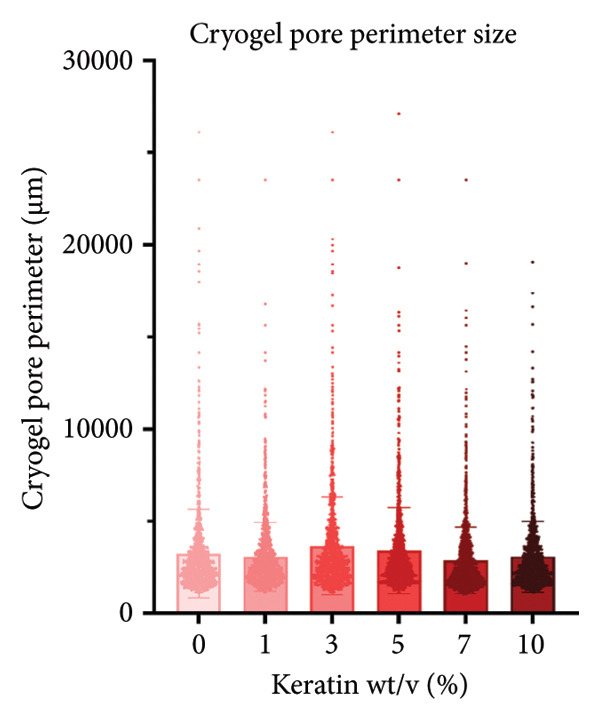
(e)

**Figure:**
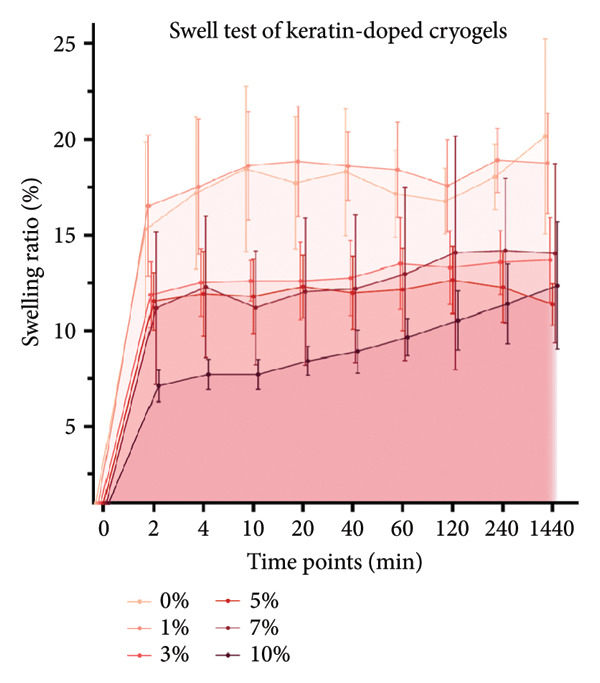
(f)

Notably, CGs experienced a slight loss in swelling capacity with increasing keratin concentration (Figure 2(f)). This was confirmed using 2‐way ANOVA with matching by the time factor and correction via Tukey’s test. There was significance between 0 and 10 wt/v% for all time points until the 24‐h measurement and significance between 0 and 5 wt/v% for all time points after 40 min. It also reported a significant difference between 0 and 3 wt/v% at the 4‐h time point (*p* < 0.05).

### 3.2. Mechanical Testing

#### 3.2.1. ESF Tensile Testing

For the vertically (Figure 3(a)) and laterally (Figure 3(b)) stretched fibers, a 1‐way Brown–Forsythe with Welch’s ANOVA test without correction for multiple comparisons (*p* < 0.05) was implemented to examine the ultimate tensile strength of the vertical (Figure 3(c)) and lateral (Figure 3(d)) fibers. When stretched until failure, vertical fibers exhibited no significant changes in tensile strength across the different keratin groups (Fig. C). For lateral fibers, increasing keratin resulted in a significantly increased ultimate tensile strength for Groups 3 and 7 compared to the 0 wt/wt% keratin group (Fig. D).

Figure 3Tensile data for ESFs (*N* = 3) showing total fiber behavior under stress for (a) vertically and (b) laterally aligned fibers. (c) The ultimate tensile strength, or point of scaffold failure, for vertical fibers showing no significant difference across the experimental groups based on a 1‐way Brown–Forsythe with Welch’s ANOVA test without correction for multiple comparisons (*p* < 0.05). (d) Measurements of laterally aligned fibers reported a general trend of increasing ultimate tensile strength with increasing keratin, with 10 wt/wt% as a trend outlier. Tensile moduli taken at the elastic deformation (straight) point on the stress/strain s‐curve for (e) vertical and (f) lateral samples. The elastic modulus taken uniformly at 10% strain for all (g) vertical and (h) lateral samples assessed with Brown–Forsythe and Welch ANOVA tests without corrections for multiple comparisons (*p* < 0.05).

**Figure:**
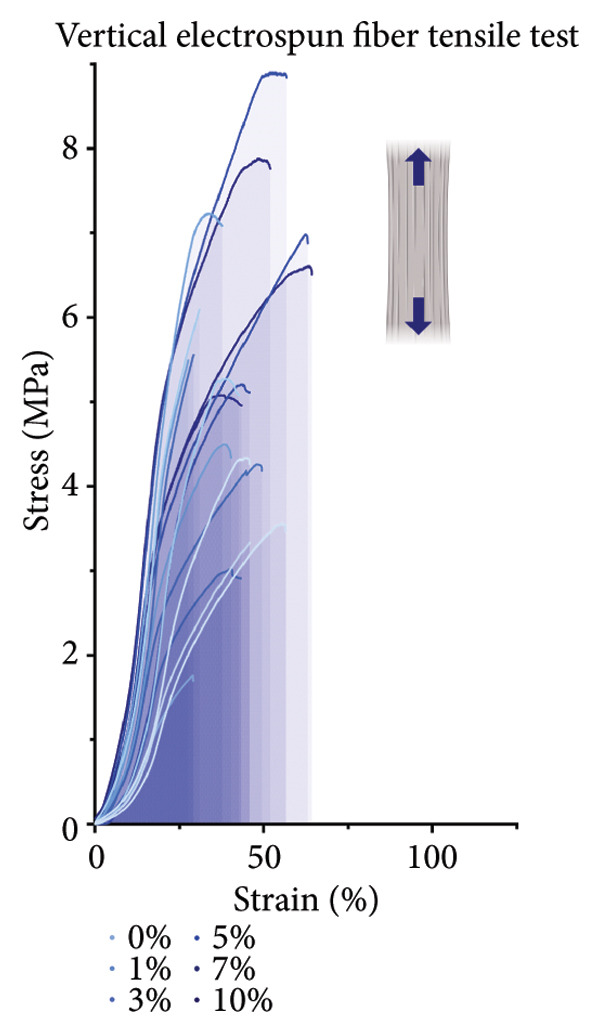
(a)

**Figure:**
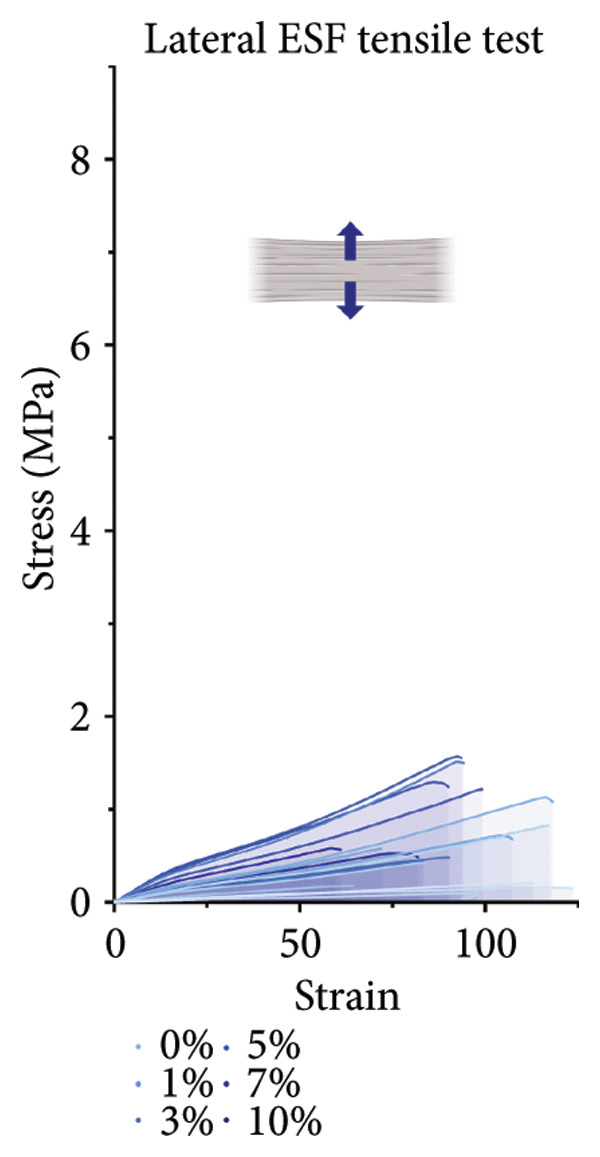
(b)

**Figure:**
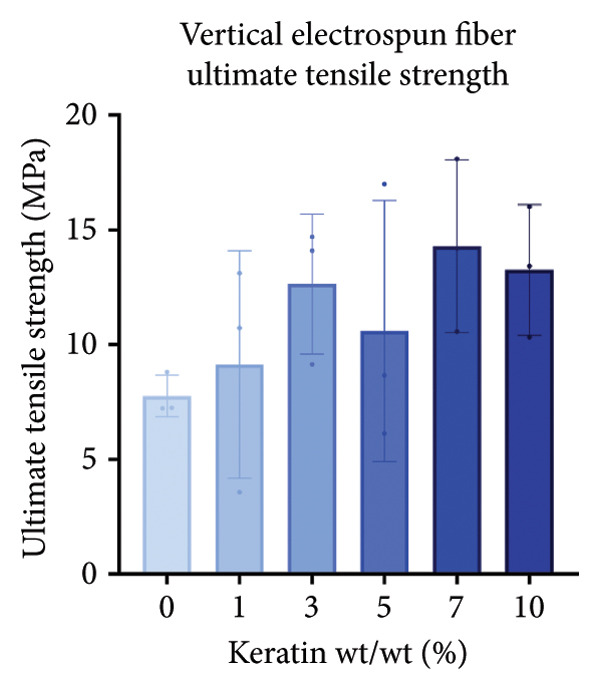
(c)

**Figure:**
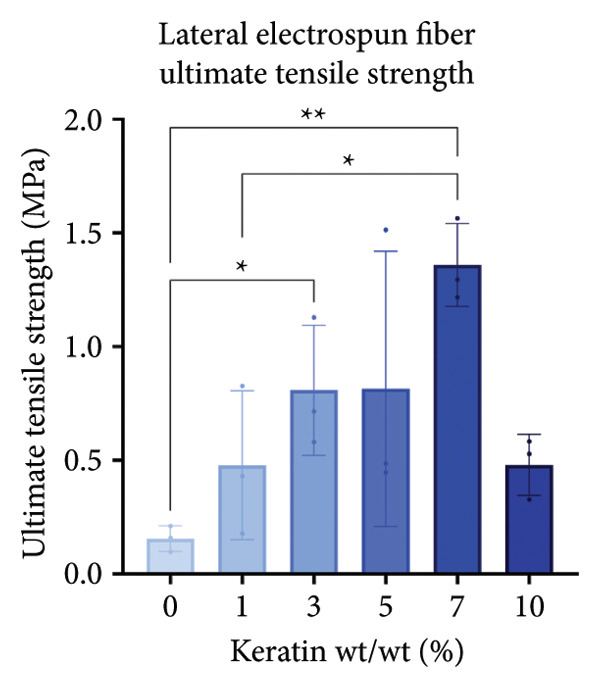
(d)

**Figure:**
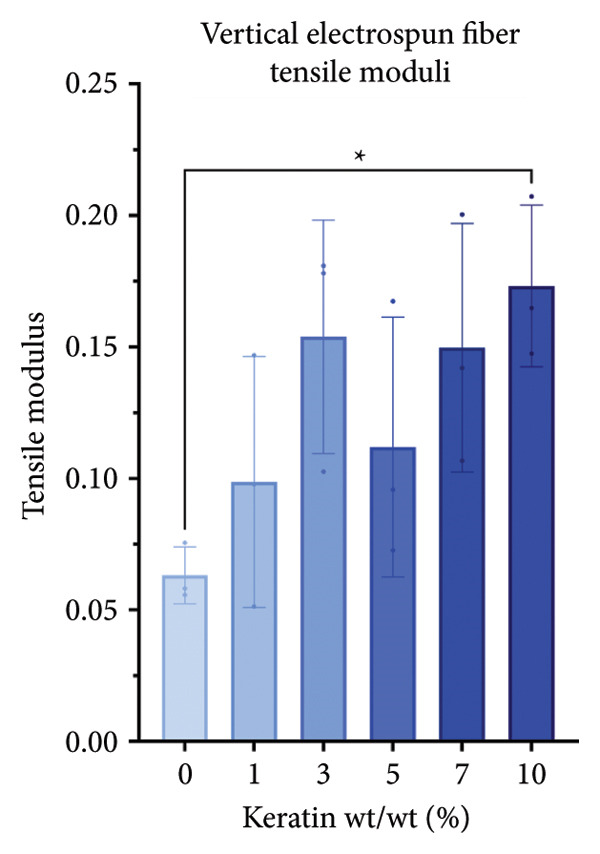
(e)

**Figure:**
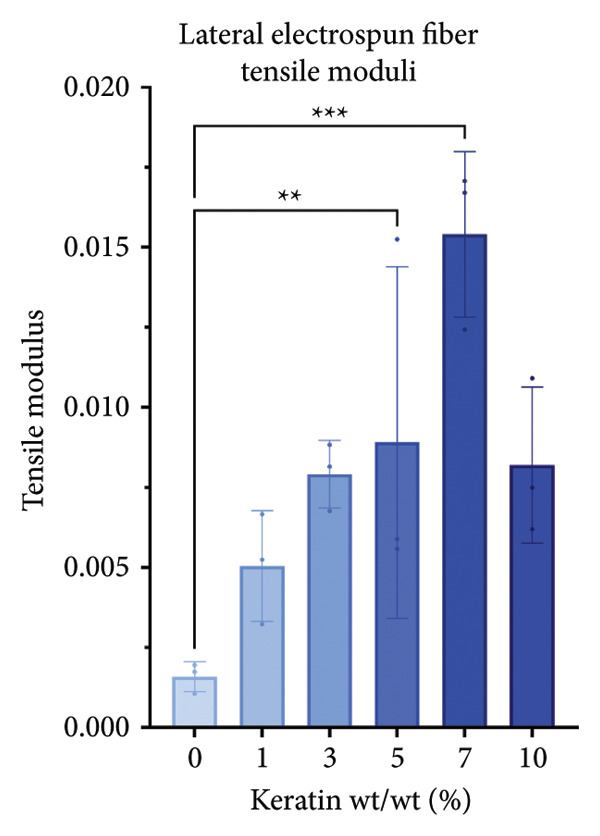
(f)

**Figure:**
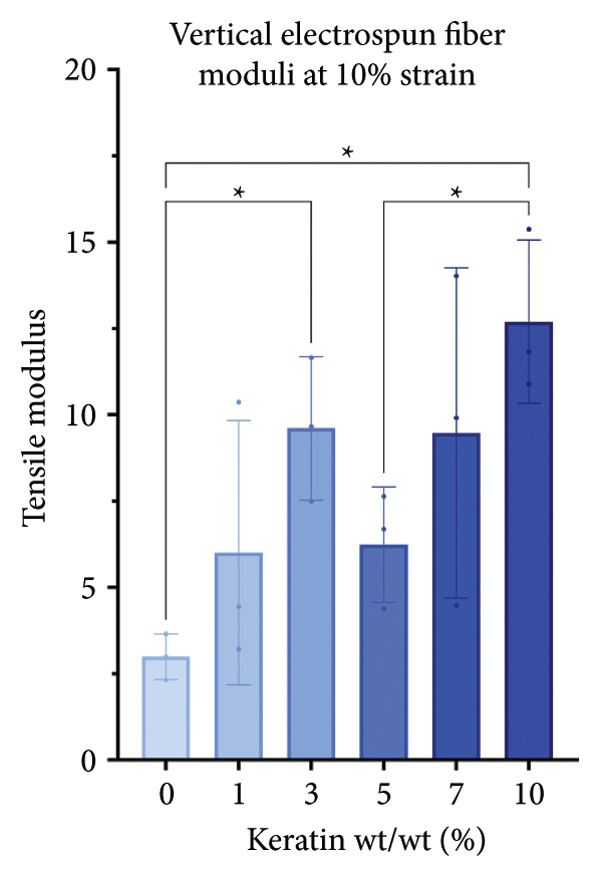
(g)

**Figure:**
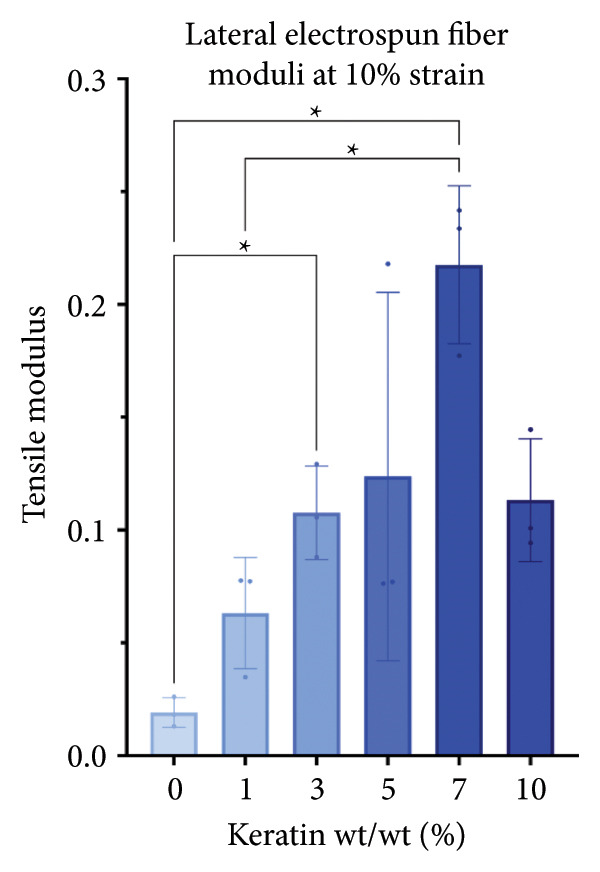
(h)

Tensile modulus measurements of the vertical fibers (Figure 3(e); *p* < 0.05) showed significance only between the 0 and 10 wt/wt% groups, indicating that the 10 wt/wt% group had a higher elastic tensile modulus compared to the 0 wt/wt% group. For the lateral fibers, both the 5 and 7 wt/wt% groups had significantly stronger tensile moduli, whereas the 10 wt/wt% group did not. This suggests that 10 wt/wt% keratin may be the point of oversaturation for the ESFs, whereas keratin under this limit provides additional binding strength between aligned fibers.

Lastly, the modulus at 10% strain was taken for both vertical and lateral fibers. For vertical fibers (Figure 3(g)), the 10% modulus revealed significant differences between the keratin concentrations (wt/wt%) of 1 and 3, 1 and 10, and 5 and 10 (Brown–Forsythe and Welch’s ANOVA tests without corrections for multiple comparisons, *p* < 0.05). For the laterally stretched fibers (Figure 3(h)), a 1‐way Brown–Forsythe with Welch’s ANOVA test without correction for multiple comparisons reported significance (*p* < 0.05) between the 0 and 7 wt/wt% group and the 1 and 7 wt/wt% group, indicating that the 7 wt/wt% group has a higher elastic tensile modulus than either the 0 or 1 wt/wt% group (Figure 3(g)). There is also a visual trend of increasing elastic modulus with increasing keratin for the laterally stretched fibers, until the 10 wt/wt% group, which is consistent with the same trend in the lateral ESF moduli in Figure 3(f).

This trend is further supported by the elastic modulus taken uniformly at 10% strain (Figure 3(h)), which shows increasing lateral modulus with increasing keratin from 0 through 7 wt/wt% (*p* < 0.05) via the Brown–Forsythe and Welch ANOVA tests without corrections for multiple comparisons.

#### 3.2.2. CG Compression Testing

For compression testing of the CGs, a stress–strain graph with all the collected Instron data (Figure 4(a)) shows a strong visual trend of decreasing compressive strength with increasing keratin concentration. This trend can also be seen in the reported ultimate tensile stresses of the CGs taken at 90% compression (Figure 4(b)) as well as in the 10% compression moduli (Figure 4(c)). Ultimate tensile stresses for keratin‐doped CGs significantly decreased when comparing 0 wt/v% to Groups 5, 7, and 10 wt/v% when using a 1‐way Brown–Forsythe with Welch’s ANOVA test with the Dunnett T3 correction for multiple comparisons. The 10% compression moduli followed this general trend with more significant decreases in tensile strength with increasing keratin. However, this decreasing trend is disrupted when the modulus is calculated at only 50% compression (Figure 4(d)), suggesting that after the initial elastic region is collapsed, the plastic deformation is more complicated to characterize. Consistent across the measurements, 0% keratin CGs have significantly higher mechanical strength.

Figure 4Compression data for CGs (*N* = 3) showing (a) total gel behavior under compressive stress for all experimental groups. (b) The ultimate tensile stress (UTS), or point of scaffold failure, for vertical fibers showing a trend of decreasing UTS with increasing keratin. (c) The compressive modulus taken uniformly at 10% strain showing significant decreases in modulus with increasing keratin. (d) The compressive modulus taken uniformly at 50% strain showing a significant decrease in modulus for all CGs with any keratin additive.

**Figure:**
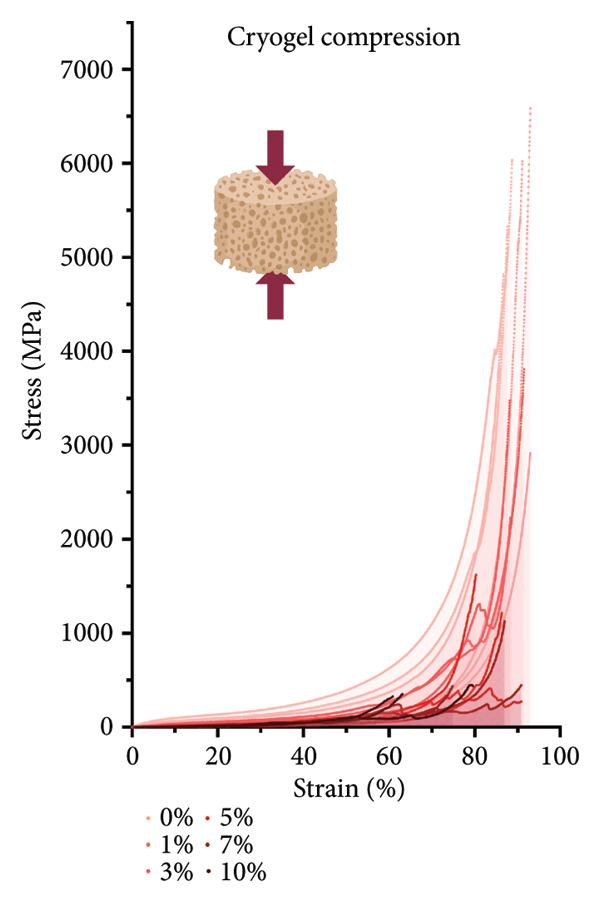
(a)

**Figure:**
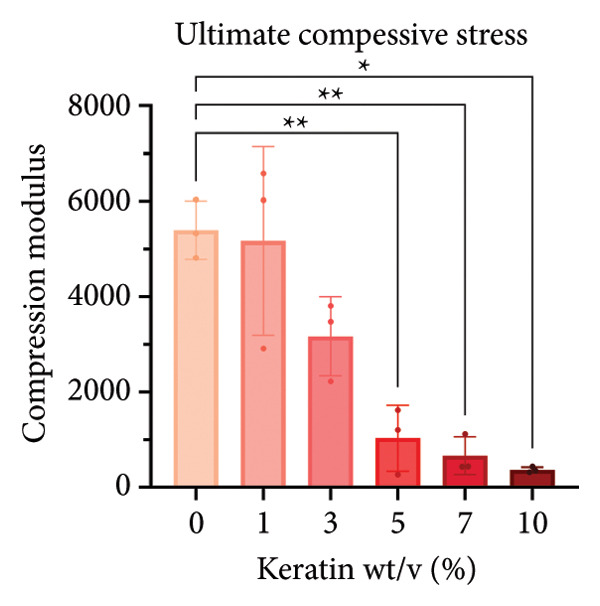
(b)

**Figure:**
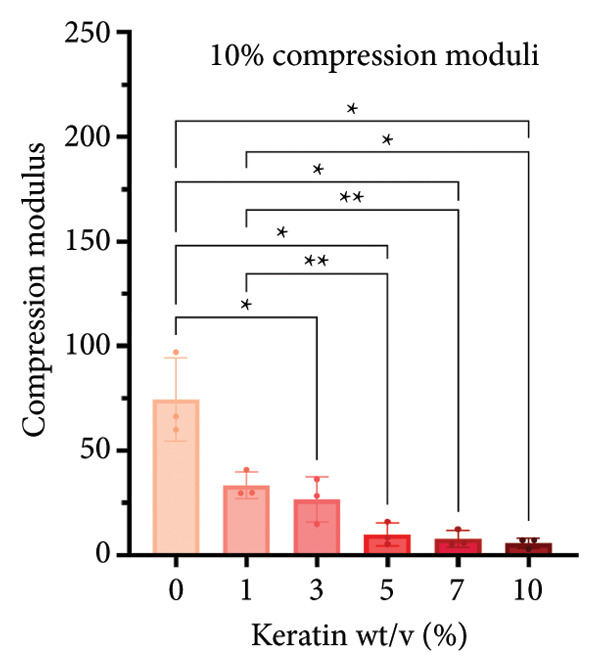
(c)

**Figure:**
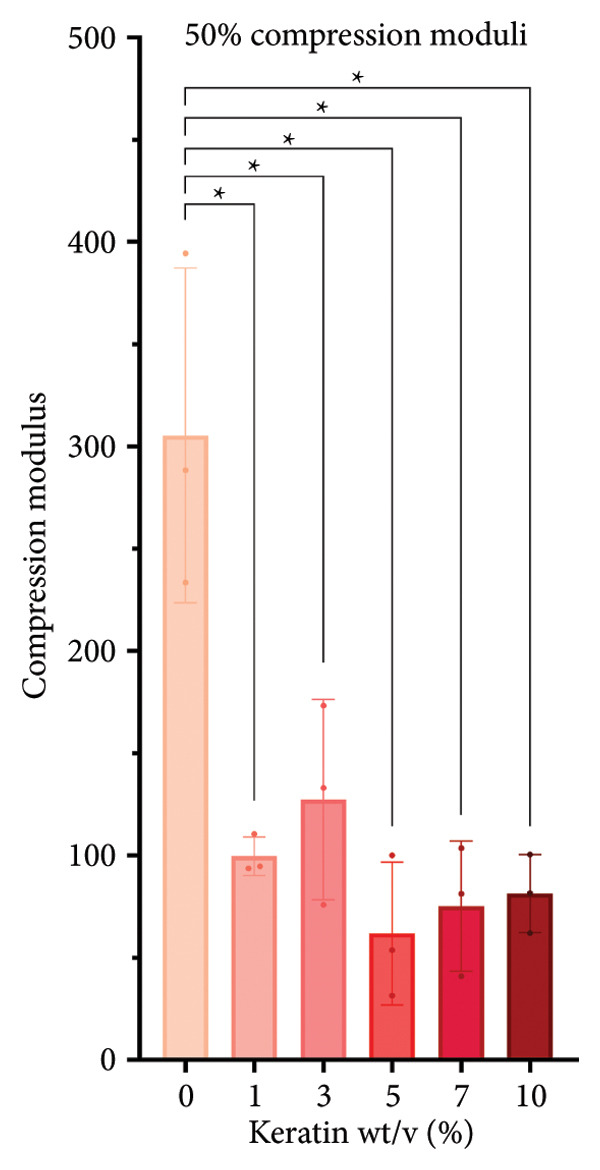
(d)

### 3.3. Degradation and Leach Testing

A degradation study was performed to assess scaffold structure and mass loss after 7‐ and 14‐day incubations in media (*N* = 4). Potential cytotoxic solute release by the keratin‐doped scaffolds was assessed via a 24‐h scratch assay in 72‐h scaffold‐conditioned media (*N* = 3).

#### 3.3.1. ESF Degradation and Leach Testing

Degradation assessments for the 7‐ and 14‐day groups were evaluated with a two‐way ANOVA full model with multiple comparisons. Representative SEM images of the scaffold surfaces at Days 7 and 14 (Figure 5(a)) show little change in visible morphology aside from some interesting aggregation of fibers in the 0 wt/wt% at Day 14. Comparatively large fibers in the 0 and 3 wt/wt% groups at Day 7 and the 5 wt/wt% group at Day 14 were also noted. These could be indicators of degradation or crosslinking of the fibers in the media. Statistical analysis of the scaffold masses (Figure 5(b)) revealed a significant increase in mass for the 1 wt/wt% keratin group both compared to the other groups within the Day 14 collection and compared to the average 1 wt/wt% mass taken on Day 7. Scratched cells incubated in keratin‐doped ESF‐conditioned media for 24 h (represented in Figure 5(c)) resulted in closures with no significant differences across the keratin percentage and control groups with a one‐way ANOVA (Figure 5(d)).

Figure 5ESF degradation study showing (a) representative SEM images of scaffolds at Days 7 and 14. (b) Mass change values for scaffolds after 7‐ and 14‐day incubations showing significant changes at Day 14 by two‐way ANOVA full model with Tukey’s correction and multiple comparisons (*p* < 0.05) (*N* = 4). (c) Representative wide‐field microscopy images of HDFas in 72‐h scaffold‐conditioned media at 0 and 24 h (*N* = 3). (d) Scratch assay results showing no significant difference among cell ability to close a model wound when exposed to conditioned compared to unconditioned media confirmed with a one‐way multiple comparisons ANOVA with the Brown–Forsythe and Welch tests (*p* < 0.05) (*N* = 3). CG degradation study showing (e) representative SEM images of scaffolds at Days 7 and 14. (f) Mass change values for scaffolds incubated in media for 7 and 14 days showing a significant decrease in mass for 10 wt/v% CGs both across the 14‐day time point and between 7 and 14 days. Day 14 data also confirm a decreasing swelling capability with increasing keratin through a two‐way ANOVA full model with Tukey’s correction and multiple comparisons (*p* < 0.05) (*N* = 4). (g) Wide‐field microscopy images of HDFa in 72‐h CG scaffold‐conditioned media and control unconditioned media at 0 and 24 h (*N* = 3). (h) Scratch assay results showing no significant difference among cell ability to close a model wound when exposed to media conditioned with scaffolds with varying keratin concentrations and a negative control of unconditioned media with a one‐way multiple comparisons ANOVA with the Brown–Forsythe and Welch tests (*p* < 0.05) (*N* = 3).

**Figure:**
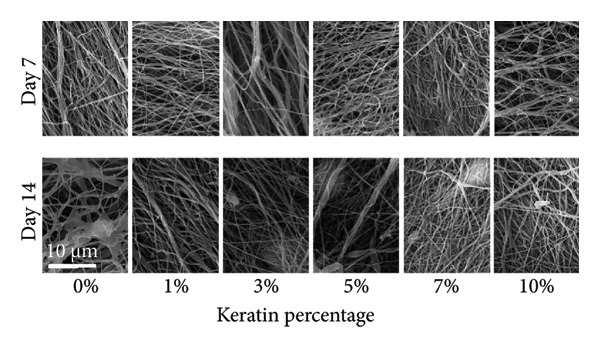
(a)

**Figure:**
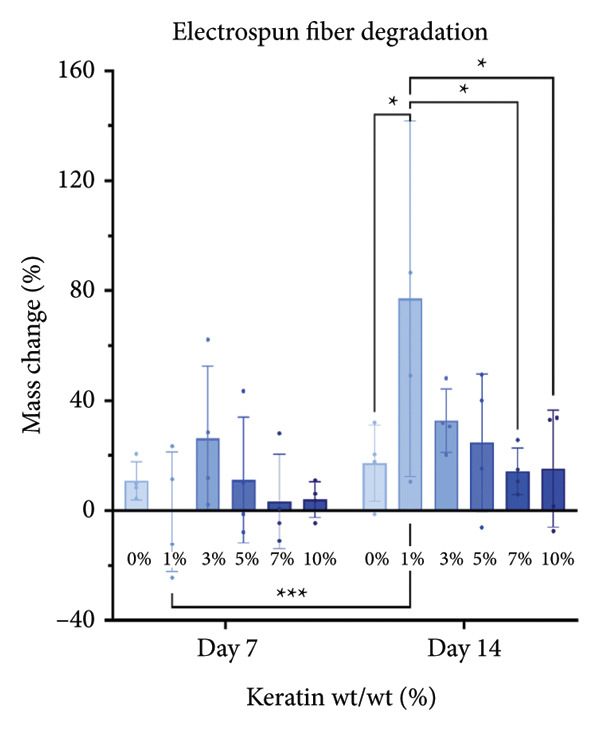
(b)

**Figure:**
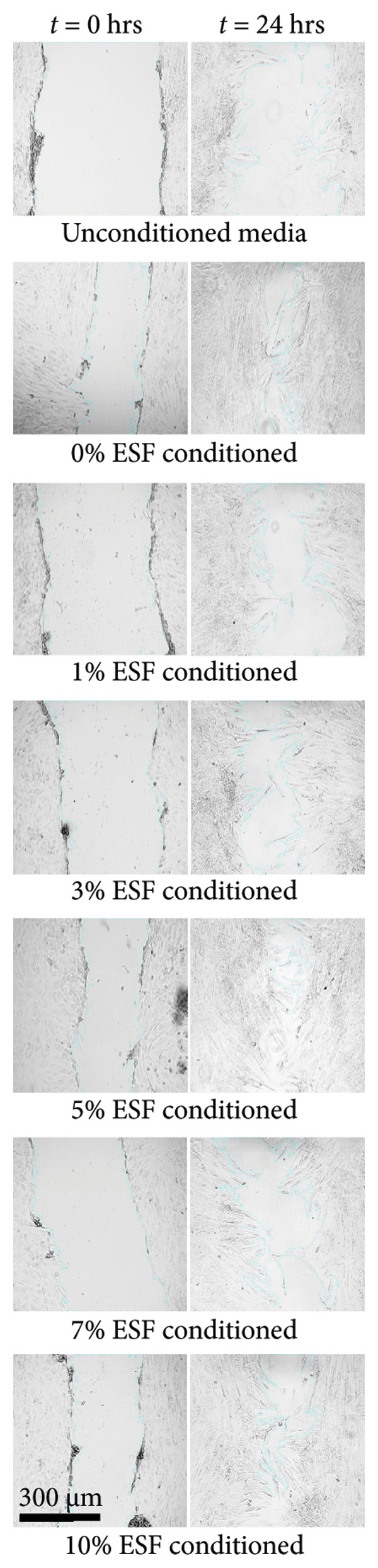
(c)

**Figure:**
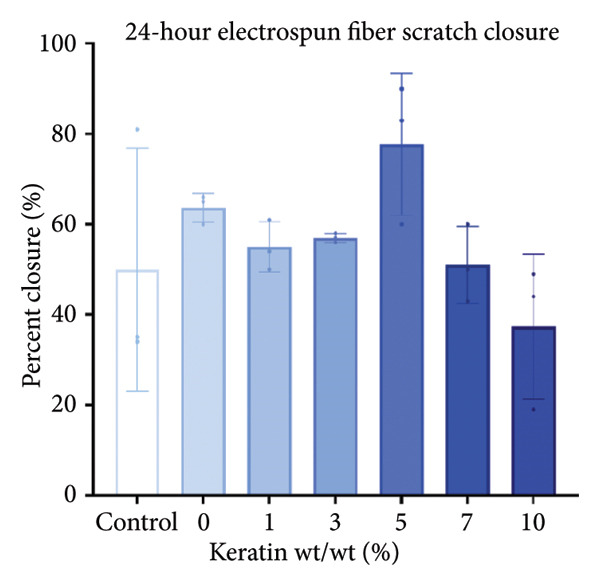
(d)

**Figure:**
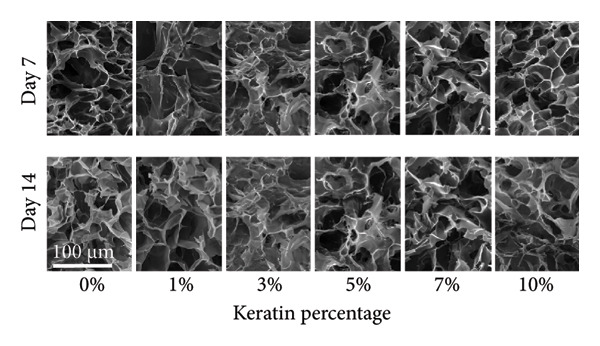
(e)

**Figure:**
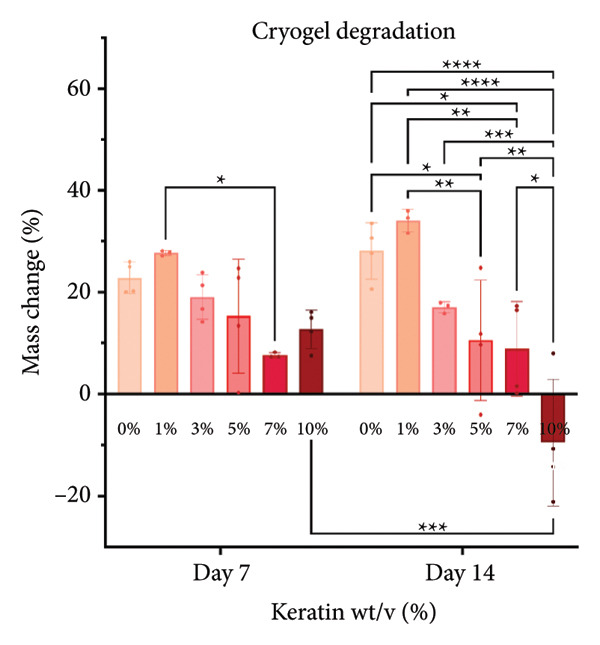
(f)

**Figure:**
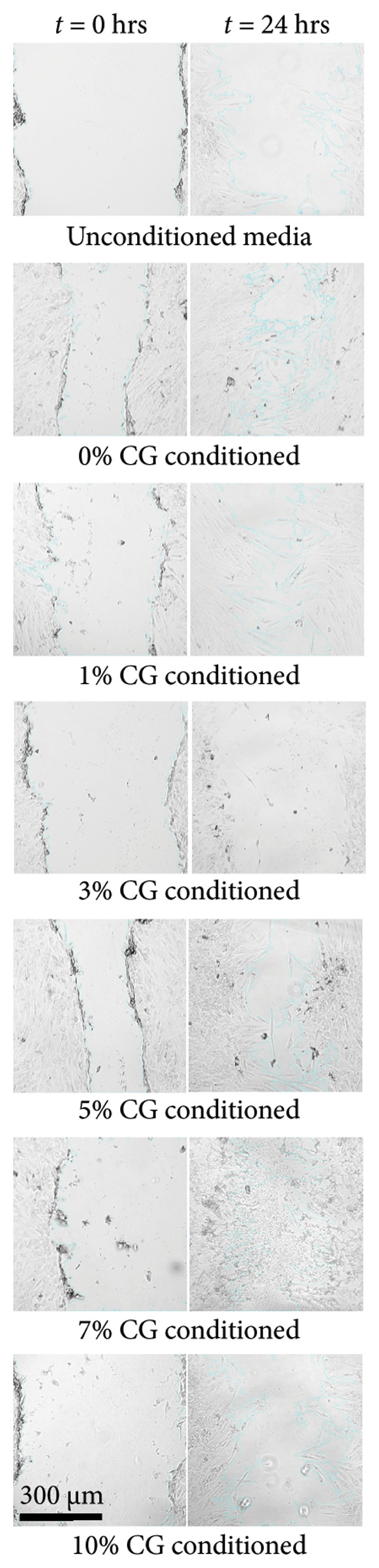
(g)

**Figure:**
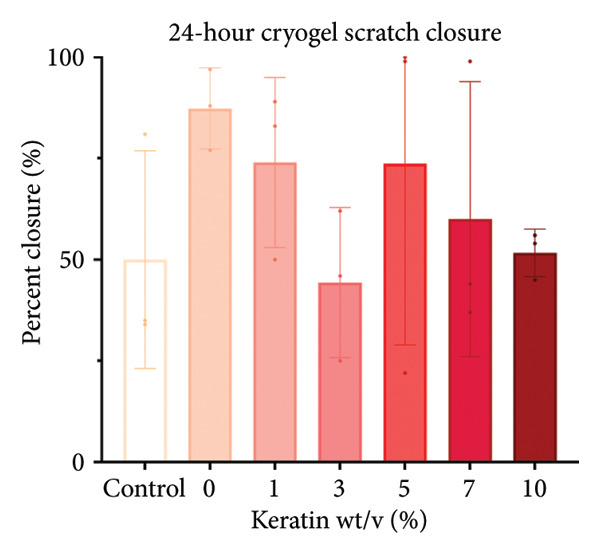
(h)

#### 3.3.2. CG Degradation and Leach Testing

For CG degradation assessment, no loss in structure of the CGs can be visually observed in the SEM images (Figure 5(e)), and their compositions are uniform and appear unaffected by the media incubation. However, 10 wt/v% CGs exhibited significant mass loss (Figure 5(f)) after 14 days of incubation in media confirming degradation and poor structural stability with high levels of keratin content. Although masses of 5 and 7 wt/v% were also significantly different from 0, 1, and 3 wt/v%, both these gels still weighed more than their initial saturated weight. This reinforces the swell test results supporting decreasing swelling potential but does not directly indicate degradation. Scratched cells incubated in keratin‐doped CG‐conditioned media (represented in Figure 5(g)) also exhibited no significant differences in closure capability across their conditioned media and scratch groups (*N* = 3) according to a one‐way ANOVA test (Figure 5(h)).

### 3.4. Cell Study

#### 3.4.1. ESF Cell Proliferation

Proliferation results (Figure 6(a)) for HDFas on ESFs did not reveal a significant trend for Days 1 or 5 but did indicate some significance at Day 3, showing a significant increase in cell presence for the 3, 7, and 10 wt/wt% ESFs (*p* < 0.05). Cell proliferation visualized by day can be found in Supporting Figure 2. Confocal images (Figure 6(b)) of stained scaffolds postseeding visibly showed HDFas attached to the scaffolds, many with long, aligned spindles displayed by the antivinculin at the 488 nm wavelength. Cells were found easily on all scaffolds and appeared well‐adhered, with active and successful adhesion sites even in the 5 and 7 wt/wt% groups at Day 7. These scaffolds appear to have experienced accelerated proliferation and begun to peel due to overcrowding. Notably, the 0 and 1 wt/wt% keratin groups show more rounded morphology and less elongated spindles, suggesting reduced adhesion capabilities at Day 7. All groups exhibited successful spread and thickening of spindle formation over the course of 7 days in culture as indicated by the green antivinculin stain.

Figure 6Cell study analysis on keratin‐doped ESFs with adult human dermal fibroblasts at Days 1, 3, and 5 postseeding. (a) Cell proliferation analysis (*N* = 3) showing scaffolds grouped by day with significant differences only for the Day 3 checkpoint between 0 and 3, 0 and 5, and 0 and 10 wt/wt%. Measured via 1‐way ANOVA with Tukey’s multiple comparisons test (*p* < 0.05). (b) Confocal images of post–cell culture scaffolds at Days 1, 3, and 7 stained with 405 nm DAPI (blue) for nuclei and a FITC antivinculin (green) for spindle adhesion showing elongated morphology with successful adhesion sites. Day 7 shows cells on 5 and 7 wt/wt% keratin scaffolds forming a confluent cell sheet and peeling off the scaffold likely due to overcrowding with the accelerated cell proliferation.

**Figure:**
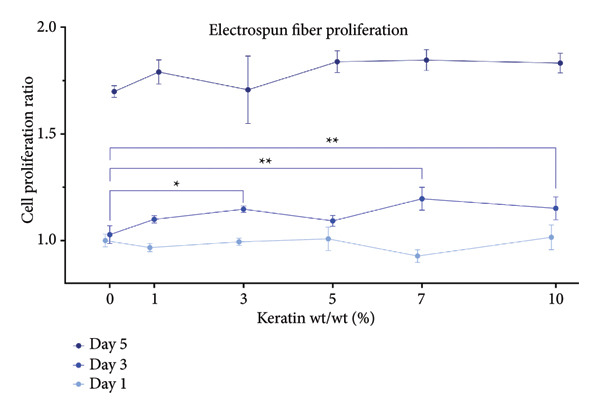
(a)

**Figure:**
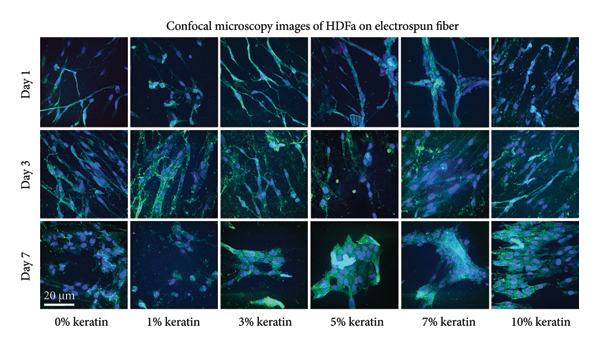
(b)

#### 3.4.2. CG Cell Proliferation

Proliferation on CG scaffolds (Figure 7(a)) increased significantly in the 3 and 5 wt/v% keratin groups by Day 5. At Day 3, only the 5 wt/v% keratin CG had significantly increased cell proliferation. Cell proliferation visualized by day can be found in Supporting Figure 3. Confocal images (Figure 7(b)) confirmed HDFa presence in all scaffolds with 405 nm DAPI. Destructive interference with an additional light channel was implemented to help counter the high fluorescence of the CG polymer material, allowing for visualization of the 405 nm emissions. Even with the additional channel, however, the 488‐nm channel was washed out by the high autofluorescence of the CG. Samples were incubated in DAPI for an additional period to allow off‐target binding and permit visualization of the cell spindles to identify continuation of cell elongation over the 7‐day culture period. Due to the 3D macroporous nature of the CG, confluence by Day 7 and peeling are not expected, as supported by the proliferation data and the confocal images, which show steadily increasing cell numbers.

Figure 7Cell study on keratin‐doped CGs with adult human dermal fibroblasts at Days 1, 3, and 5 postseeding. (a) Cell proliferation analysis (*N* = 3) showing scaffolds grouped by day with Day 1 indicating significant differences noted for 10% when compared with 0, 1, and 3 wt/v%. Day 3 showed significant differences when comparing 1 wt/v% to both 7 and 10 wt/v%. Day 5 then showed significant differences when comparing 1 wt/v% with 3, 5, and 10 wt/v% and when comparing 1 and 10 wt/v%. Significance was measured via 1‐way ANOVA comparing within‐day checkpoint groups with Tukey’s multiple comparisons test (*p* < 0.05). (b) Confocal images of post–cell culture scaffolds stained with 405 nm DAPI (blue) for the HDFa nuclei, and a FITC antivinculin (green) for spindle adhesion, which was largely overwhelmed by the cryogel’s natural autofluorescence.

**Figure:**
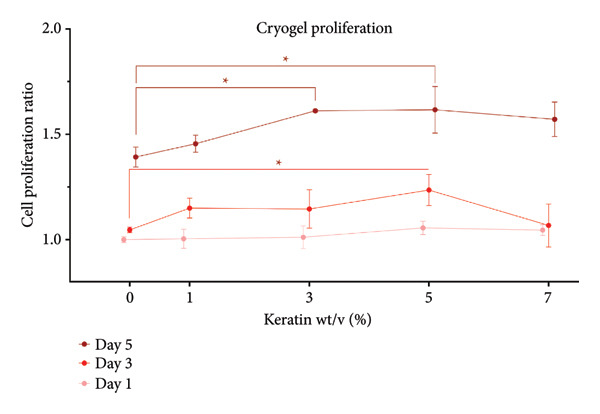
(a)

**Figure:**
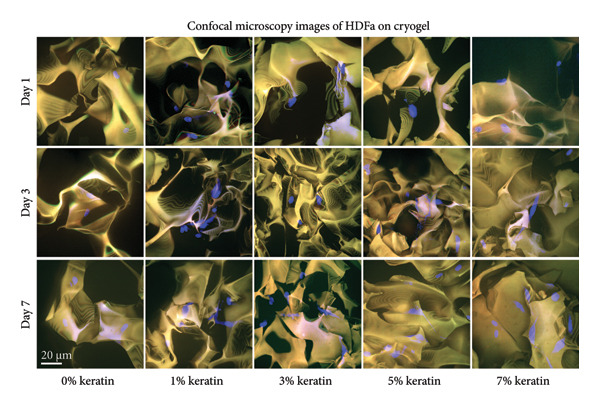
(b)

## 4. Discussion

### 4.1. Scaffold Characterization: Surface and Mechanical Analyses

Although surface analyses revealed a significant effect of keratin on the structure of both ESFs and CGs, these changes were not sufficient to preclude cell adhesion. While increasing keratin content led to a significant decrease in pore and fiber features, these alterations did not eliminate the scaffolds’ suitability for supporting cell adhesion, warranting further investigation. For ESFs, keratin generally increases the mechanical strength of the fibers in both vertical and lateral tests, which supports the use of higher keratin concentrations. This increase in strength can potentially be attributed to the relationship between increasing keratin and increasing water content in the electrospinning solution. Water evaporates slower than TFE, making high water content solutions more likely to form fibers closer to the collection barrel, potentially making them less brittle. The decreasing fiber diameters with increasing keratin also make it possible that the mesh gained the flexibility of a greater number of finer fibers. However, the 10 wt/wt% does present as an outlier for the lateral group. This could be due to a loss of alignment of the fibers as seen in the surface analysis. Here, we can see that the 10 wt/wt% ESFs do lose the majority of their directionality, which was likely due to unstable Taylor cone conditions while spinning. This inability to maintain directionality reduces the tunability of the scaffold. The reduction in tunability combined with the higher‐maintenance spinning makes the 10 wt/wt% ESFs a poor candidate for use as tissue scaffolds. For the CGs, visual assessment and compression testing did reveal that the mechanical properties of the 10 wt/v% CG were disrupted to the point that the scaffold could no longer independently hold its shape. After swelling, the scaffolds were also visually more translucent and broke apart easily even with gentle handling. This confirms that the upper limit for testing that was chosen does reach the point of nonviability as a tissue scaffold. The reduced mechanical properties would prevent the 10 wt/v% CG from being able to hold its shape, which could result in pieces of CG breaking off and migrating out of the target wound site. The general, visual trend of decreasing swelling capacity with increasing keratin is likely due to interference in the crosslinking process caused by the extra additive reducing expansion capabilities of the gel, which would likely also cause the mechanical weakness. This same phenomenon could also account for the shrinkage of scaffold pores in CGs. It is likely that the presence of the keratin additive prevents large molecular crosslinked structures from forming in the gel, shrinking the sizes of the subsequent pores formed by the water crystals. It is also possible that, due to the hydrolyzed keratin’s water‐soluble nature, the dispersion of particles in the solution is more homogenous before freezing can occur, causing the water molecules to be more evenly dispersed, which therefore results in smaller pore formation.

During contact angle recordings, there were some interesting discrepancies in the higher keratin groups. For 10 wt/wt% ESFs, two‐thirds of the fibers absorbed rapidly, whereas one‐third did not and retained minimal absorption. Additional and repeat samples were tested for the higher groups, and the pattern continued. This high variability in the 10 and, to a lesser extent, the 7 wt/wt% groups is theorized to be due, at least in part, to the poor spinning reproducibility already discussed. Especially for the 10 wt/wt% group, the frequent stopping and starting of the spin paired with partially dried or sputtering Taylor cones could have caused inconsistent fiber quality on the top layer of the fibers depending on when in the dry, pause, wipe, and restart cycle the surface fibers had been deposited. It is also possible that the keratin was not homogenously dissolved in the higher concentration groups, and so the differences were due to nonuniform regions within the fiber sheets. This variability made it difficult to determine statistical significance for the 10 wt/wt% group as its sample inconsistency was so high. Overall, the highly inconsistent surface energy with the higher wt/wt% keratin ESF groups is a disadvantage for use as tissue‐engineered scaffolds.

### 4.2. Degradation and Leach Evaluation

#### 4.2.1. ESF Degradation and Solute Leaching

SEM images of the fibers (Figure 5(a)) showed no consistent changes in fiber appearance with increasing keratin or between the two tested degradation time points. When compared with SEM images of preincubation ESFs (Figures 1(a) and 1(b)), the morphology after 7‐ and 14‐day incubation in media (Figure 5(a)) appears consistent. 1%, 7%, and 10% fibers possessed similar fiber diameters, distributions, and directionality. However, there is some shriveling of the fibers evident at both Days 7 and 10, whereas unincubated fibers are straight and smooth. The 0 wt/wt% keratin also exhibits an interesting morphology where the fibers appear to have run or crosslinked with one another after 14 days. It is also possible that this is due to batch variety, but it is interesting to note the consistency of the varying fiber diameters across the unincubated fibers in Figures 1(a) and 1(b) and the incubated fibers in Figure 5(a).

Mass loss analysis over 7 and 14 days for ESFs (Figure 5(b)) revealed no apparent degradation and did indicate substantial mass gain through absorption of the media solution as compared to the 0 wt/wt% keratin control. ESFs appeared to absorb very slowly during the initial submerging, which is likely due to their microporous composition; it takes longer for air to diffuse out of the scaffold through the tight‐knit mesh. Here, 1 wt/wt% keratin experienced a mass increase compared to the other values and the control at Day 14. Note that four different ESF scaffolds were used for this analysis with a high distribution of values. At Day 7, there was also a comparatively large distribution of mass values recorded, which suggests a high batch variance. It is possible that the 1 wt/wt% keratin contains too little keratin to provide a substantial polymer structure but is just enough to disrupt proper crosslinking of the PCL polymer, resulting in variable swelling capabilities across otherwise uniform batches.

Cell morphology and migration during the conditioned media scratch assay also appear normal and consistent in scratch images (Figure 5(c)). The scratch closure data reported no significant changes in wound closure ability for any of the ESF keratin groups (Figure 5(d)). These data indicate that no harmful solutes negatively affecting cell proliferation and migration are being released into the media from the keratin‐doped ESFs.

#### 4.2.2. CG Degradation and Solute Leaching

SEM images of the incubated CGs reveal no visual cues to detect degradation even in the 10 wt/v% (Figure 5(e)) as compared to preincubation CGs (Figures 2(a) and 2(b)); however, there are significant changes in mass evident in Figure 5(f). This suggests that degradation is occurring uniformly and in a way that does not affect the pore morphology of the CG. Mass loss analysis over 7 and 14 days for CGs (Figure 5(f)) revealed significant degradation through mass loss in the 10 wt/v% group at Day 14. CGs absorb very quickly during the initial submersion, which is likely due to their macroporous and sponge‐like composition; the pores are large, and subsequently, the CGs swell rapidly. The mass gained by the other groups reinforces data from the swelling analysis, indicating that swelling potential is generally reduced with increasing keratin concentration. This is only evident at Day 7 in the 7 wt/v% group but is shown at Day 14 in Groups 5 and 7 wt/v%. Interestingly, 10 wt/v% at Day 10 does not have a significant reduction in swelling capacity, and this is likely due to the shift toward a more hydrogel‐like consistency with 10 wt/v%, as can be seen in section Figure 2(c). This change in gel presentation also explains the mass loss at Day 14. Given the confirmed degradation in this group, the 10 wt/v% formulation was deemed unsuitable, indicating that our keratin distribution captured the upper limit of feasibility for this scaffold design. Therefore, this group was removed for the following cell study testing.

Cell morphology and migration also appear normal and consistent in scratch images represented in Figure 5(g). Corresponding scratch data reported no significant changes in wound closure ability for any of the CG keratin groups (Figure 5(h)). This indicates that no harmful solutes that affect cell proliferation and migration are being released into the media from the keratin‐doped CGs.

### 4.3. Cell and Proliferation Analyses

For ESFs, the proliferation analysis reports an early increase in cell growth by Day 3. However, there are no significant differences in cell growth among the groups by Day 7. This result can be explained by the confocal images for Day 7, which display consistent morphological cell behavior until Day 7. The change in morphology and conformation from a flat plane of dispersed and elongated cells to a tight network of folded cell sheets suggests attainment of confluency and pseudotissue formation. This can be seen in Groups 3, 5, and 7 wt/wt%, which could correspond to the early heightened proliferation seen in Groups 3 and 7 wt/wt% at Day 3. This suggests that by Day 7, all the groups are at or nearing the capacity point for the two‐dimensional (2D) ESF scaffold and those that saw faster proliferation have had their growth capped and are beginning to change conformation. The 10 wt/wt% content is potentially the oversaturation point where we see a slowing of proliferation and an earlier stage of sheet formation and peeling at Day 7 on the confocal images. However, the confocal images reveal that there seems to be visibly less directionally aligned spread to the cells in the higher 7 and 10 wt/wt% groups. This resulted in more sporadic adhesion sites and unaligned cell behavior, which is not consistent with healthy HDFa spreading. When combined with the poorly aligned fibers seen in the SEM images, this indicates that the scaffolds with higher concentrations may be less suited for rapid HDFa adhesion. However, it is interesting to note that the 0 wt/wt% keratin groups also exhibited limited alignment despite the highly aligned fibers noted in the SEM images.

For GCs, cell proliferation results demonstrate increased proliferation with the middle concentrations of keratin (3 and 5 wt/v%) by Day 5. This is somewhat consistent with Day 3 data showing only the 5 wt/v% group as significantly promoting proliferation. Confocal images support this trend with 3 wt/v% showing fewer cells at Day 3 than the 5 wt/v% group before catching up by Day 7 to show cell numbers consistent with 5 wt/v%. Interestingly, the 7 wt/v% group also shows a greater number of cells than Groups 0 and 1 wt/v% at Day 7, resembling Groups 3 and 5 wt/v% despite showing a lower proliferation value. It is possible that the cells react poorly to the decreasing mechanical strength of the 7 wt/v% CG or that the pore size decrease in the 7 wt/v% group was greater than that of the 3% and 5% groups as the statistical analysis revealed, and this did in fact began to hinder cell migration despite our previous hypothesis. Due to the highly complex and 3D nature of the CG scaffold, there also appear to be fewer cells when imaging as they have more room to migrate and the pores obscure the cells that have penetrated deeper into the scaffold. However, repeated proliferation data and consistent cell distribution on confocal images support the reported number of cells in this study.

## 5. Conclusion

This study provides a comprehensive examination of the effects of hydrolyzed keratin on CGs and ESFs. It was hypothesized that hydrolyzed keratin would provide additional binding sites for cells and would thereby increase the cell adhesion without significantly compromising the desirable scaffold properties. This work suggests that although hydrolyzed keratin does have a positive effect on cell proliferation in the scaffold, the higher concentrations tested did have adverse effects on the scaffold properties. For the ESFs, the highest keratin concentration tested (10 wt/wt%) resulted in poor fiber alignment due to unstable spinning conditions and required frequent stop–start. At 7 wt/wt%, alignment and reproducibility were more variable, reflecting increased sensitivity of the spinning process at elevated keratin content. For CGs, higher keratin concentration resulted in unstable gels and poor cell adhesion (10 wt/v%). Therefore, based on the data, it is advisable to proceed with the moderately keratin‐doped scaffolds.

For ESFs, 5 and 7 wt/wt% keratin/PCL scaffolds represent the most promising samples. These groups exhibited higher proliferation at the Day 3 checkpoint, while 7 wt/wt% required tighter control of spinning parameters, both concentrations were capable of maintaining a stable Taylor cone under optimized conditions. The fibers were also aligned, and all cells at Days 1 and 3 appeared healthy and well‐spread. These groups also had a high tensile strength and elasticity, which would have high potential in a tissue engineering setting with the option for either a scaffold with a lower (5 wt/wt%) or higher (7 wt/wt%) surface energy depending on the desired cell adhesion and protein adsorption. For CGs, scaffolds fabricated with 3 and 5 wt/v% keratin/chitosan–gelatin solution demonstrated the best performance. These scaffold groups had high proliferation without drastically sacrificing mechanical strength. Confocal images also revealed promising clustering and ingrowth into the CG topography for these groups. Overall, although the keratin additive had a more modest effect on cell proliferation and adhesion than anticipated, the results demonstrate a clear positive impact at early time points, particularly in CG scaffolds, where evidence suggests an acceleration of cellular interactions. Moreover, the observed effects on scaffold properties highlight opportunities for tailoring constructs to optimize performance in soft tissue applications.

## Conflicts of Interest

The authors declare no conflicts of interest.

## Funding

This study was supported by the Hitchcock Foundation (Dartmouth Health), the National Institutes of Health (NIH), National Institute for Biomedical Imaging and Bioengineering (NIBIB) T32 Training in Surgical Innovation Program (5T32EB021966‐07), and bioMT through NIH (P20‐GM113132).

## Supporting Information

Supporting Figure 1: Expanded contact angle analysis (*N* = 3) showing a slight increase in contact angle with low keratin concentrations followed by a rapid and inconsistent drop in contact angle in the 7 and 10 wt/wt% groups. Detailed significance between the groups is from a 2‐way ANOVA with Tukey’s correction for multiple comparisons with a single pooled variance test.

Supporting Figure 2: Cell analysis showing scaffolds grouped by percentage with proliferation magnitude over Days 1, 3, and 5 represented in a stacked bar. Cell proliferation analysis showing scaffolds grouped by day with significant differences only for the Day 3 checkpoint between 0 and 3, 0 and 5, and 0 and 10 wt/wt%. Data were measured via 1‐way ANOVA with Tukey’s multiple comparisons test (*p* < 0.05).

Supporting Figure 3: Cell analysis showing scaffolds grouped by percentage with proliferation magnitude over Days 1, 3, and 5 represented in a stacked bar. Day 1 indicates significant differences noted for 10% when compared with 0, 1, and 3 wt/v%. Day 3 showed significant differences when comparing 1 wt/v% to both 7 and 10 wt/v%. Day 5 then showed significant differences when comparing 1 wt/v% with 3, 5, and 10 wt/v% and when comparing 1 and 10 wt/v%. Significance was measured via 1‐way ANOVA comparing within‐day checkpoint groups with Tukey’s multiple comparisons test (*p* < 0.05).

## Supporting information


**Supporting Information** Additional supporting information can be found online in the Supporting Information section.

## Data Availability

The data that support the findings of this study are available from the corresponding author upon reasonable request.

## References

[1] Bumbaširević M. , Lesic A. , Palibrk T. et al., The Current State of Bionic Limbs From the Surgeon’s Viewpoint, EFORT Open Reviews. (2020) 5, no. 2, 65–72, 10.1302/2058-5241.5.180038. 32175092 PMC7047902

[2] Brånemark P. I. , Osseointegration and Its Experimental Background, The Journal of Prosthetic Dentistry. (1983) 50, no. 3, 399–410, 10.1016/s0022-3913(83)80101-2, 2-s2.0-0020823258. 6352924

[3] Engida A. , Ayelign T. , Mahteme B. , Aida T. , and Abreham B. , Types and Indications of Colostomy and Determinants of Outcomes of Patients After Surgery, Ethiop J Health Sci. (2016) 26, no. 2, 117–120, 10.4314/ejhs.v26i2.5. 27222624 PMC4864340

[4] Black G. G. , Vaeth A. M. , Chen Y. et al., 86. 5 Years of Lower Limb Osseointegrated Prostheses: Anticipating the “Who and What” of Poor Outcomes, Plastic and Reconstructive Surgery: Global Open. (2023) 11, no. 5S, 10.1097/01.GOX.0000937936.23076.56.

[5] Ambe P. C. , Kurz N. R. , Nitschke C. , Odeh S. F. , Möslein G. , and Zirngibl H. , Intestinal Ostomy: Classification, Indications, Ostomy Care and Complication Management, Dtsch Arztebl Int. (2018) 115, no. 11, 182–187, 10.3238/arztebl.2018.0182, 2-s2.0-85044987563. 29607805 PMC5913578

[6] Cheung E. , Baerlocher M. O. , Asch M. , and Myers A. , Venous Access: A Practical Review for 2009, Canadian Family Physician. (2009) 55, no. 5, 494–496. 19439704 PMC2682308

[7] Tropf J. G. and Potter B. K. , Osseointegration for Amputees: Current State of Direct Skeletal Attachment of Prostheses, Orthopaedic Surgery. (2023) 12, no. C, 20–28, 10.1016/j.orthop.2023.05.004.

[8] Abdallah M. , Badran Z. , Ciobanu O. , Hamdan N. , and Tamimi F. , Strategies for Optimizing the Soft Tissue Seal Around Osseointegrated Implants, Advanced Healthcare Materials. (2017) 6, no. 20, 10.1002/adhm.201700549, 2-s2.0-85030308811. 28960892

[9] Chen H. , Agrawal D. K. , and Thankam F. G. , Biomaterials-Driven Sterile Inflammation, Tissue Engineering Part B Reviews. (2022) 28, no. 1, 22–34, 10.1089/ten.TEB.2020.0253. 33213285 PMC8892963

[10] Svensson S. , Trobos M. , Hoffman M. et al., A Novel Soft Tissue Model for Biomaterial-Associated Infection and Inflammation: Bacteriological, Morphological and Molecular Observations, Biomaterials. (2015) 41, 106–121, 10.1016/j.biomaterials.2014.11.032, 2-s2.0-84916880350. 25522970

[11] Li H. , Zhang S. , Huo S. et al., Effects of Staphylococcal Infection and Aseptic Inflammation on Bone Mass and Biomechanical Properties in a Rabbit Model, J Orthop Translat. (2019) 21, 66–72, 10.1016/j.jot.2019.11.006. 32099806 PMC7029375

[12] Mcgrath J. A. and Uitto J. , Rook′s Textbook of Dermatology, 2010, Wiley-Blackwell.

[13] Proksch E. , Brandner J. M. , and Jensen J. M. , The Skin: An Indispensable Barrier, Experimental Dermatology. (2008) 17, no. 12, 1063–1072, 10.1111/j.1600-0625.2008.00786.x, 2-s2.0-56349089597. 19043850

[14] Abdallah M. N. , Badran Z. , Ciobanu O. , Hamdan N. , and Tamimi F. , Strategies for Optimizing the Soft Tissue Seal Around Osseointegrated Implants, Advanced Healthcare Materials. (2017) 6, no. 20, 10.1002/adhm.201700549, 2-s2.0-85030308811. 28960892

[15] Gurtner G. , Werner S. , Barrandon Y. , and Longaker M. T. , Wound Repair and Regeneration, Nature. (2008) 453, no. 7193, 314–321, 10.1038/nature07039, 2-s2.0-43749088730. 18480812

[16] Pendegrass C. J. , Goodship A. E. , and Blunn G. W. , Development of a Soft Tissue Seal Around Bone-Anchored Transcutaneous Amputation Prostheses, Biomaterials. (2006) 27, no. 23, 4183–4191, 10.1016/j.biomaterials.2006.03.041, 2-s2.0-33646141601. 16618500

[17] Overmann A. L. and Forsberg J. A. , The State of the Art of Osseointegration for Limb Prosthesis, Biomed Eng Lett. (2019) 10, no. 1, 5–16, 10.1007/s13534-019-00133-9. 32175127 PMC7046912

[18] Weigel T. , Christ B. , Dembski S. et al., Biomimetic Connection of Transcutaneous Implants With Skin, Advanced Healthcare Materials. (2023) 12, no. 30, 10.1002/adhm.202301131. PMC11469180 37660290

[19] Fitzpatrick N. , Smith T. J. , Pendegrass C. J. et al., Intraosseous Transcutaneous Amputation Prosthesis (ITAP) for Limb Salvage in 4 Dogs, Veterinary Surgery. (2011) 40, no. 8, 909–925, 10.1111/j.1532-950X.2011.00891.x, 2-s2.0-84863995486. 22092391

[20] Kang N. V. , Pendegrass C. , Marks L. , and Blunn G. , Osseocutaneous Integration of an Intraosseous Transcutaneous Amputation Prosthesis Implant Used for Reconstruction of a Transhumeral Amputee: Case Report, Journal of Hand Surgery. (2010) 35, no. 7, 1130–1134, 10.1016/j.jhsa.2010.03.037, 2-s2.0-77954086207. 20541327

[21] Chimutengwende-Gordon M. , Pendegrass C. , and Blunn G. , The in Vivo Effect of a Porous Titanium Alloy Flange With Hydroxyapatite, Silver and Fibronectin Coatings on Soft-Tissue Integration of Intraosseous Transcutaneous Amputation Prostheses, Bone & Joint J. (2017) 99, no. 3, 393–400, 10.1302/0301-620X.99B3.BJJ-2016-0360.R1, 2-s2.0-85017558431. 28249981 PMC5358203

[22] Brahs A. B. and Bolla S. R. , Histology, Nail, 2024, StatPearls Publishing, https://www.ncbi.nlm.nih.gov/books/NBK539733/. 30969555

[23] Vielmuth F. , Wanuske M. T. , Radeva M. Y. et al., Keratins Regulate the Adhesive Properties of Desmosomal Cadherins Through Signaling, Journal of Investigative Dermatology. (2018) 138, no. 1, 121–131, 10.1016/j.jid.2017.08.033, 2-s2.0-85039948174. 28899688

[24] Soleymani Eil Bakhtiari S. and Karbasi S. , Keratin-Containing Scaffolds for Tissue Engineering Applications: A Review, Journal of Biomaterials Science. (2024) 35, no. 6, 916–965, 10.1080/09205063.2024.2311450. 38349200

[25] Rouse J. G. and Van Dyke M. E. , A Review of Keratin-Based Biomaterials for Biomedical Applications, Materials. (2010) 3, no. 2, 999–1014, 10.3390/ma3020999, 2-s2.0-77956092359.

[26] Vielmuth F. , Wanuske M. T. , Radeva M. Y. et al., Keratins Regulate the Adhesive Properties of Desmosomal Cadherins Through Signaling, Journal of Investigative Dermatology. (2018) 138, no. 1, 121–131, 10.1016/j.jid.2017.08.033, 2-s2.0-85039948174. 28899688

[27] Bhat H. F. , Amin N. , Nasir Z. et al., Keratin as an Effective Coating Material for *in Vitro* Stem Cell Culture, Induced Differentiation and Wound Healing Assays, Heliyon. (2024) 10, no. 5, 10.1016/j.heliyon.2024.e27197. PMC10923720 38463859

[28] Kathuria N. , Tripathi A. , Kar K. , and Kumar A. , Synthesis and Characterization of Elastic and Macroporous Chitosan–gelatin Cryogels for Tissue Engineering, Acta Biomaterialia. (2009) 5, no. 1, 406–418, 10.1016/j.actbio.2008.07.009, 2-s2.0-56349113678. 18701361

[29] Khandaker M. , Nomhwange H. , Progri H. , Nikfarjam S. , and Vaughan M. B. , Evaluation of Polycaprolactone Electrospun Nanofiber-Composites for Artificial Skin Based on Dermal Fibroblast Culture, Bioengineering. (2022) 9, no. 1, 10.3390/bioengineering9010019. PMC8773077 35049727

[30] Li W. J. , Laurencin C. T. , Caterson E. J. , Tuan R. S. , and Ko F. K. , Electrospun Nanofibrous Structure: A Novel Scaffold for Tissue Engineering, Journal of Biomedical Materials Research. (2002) 60, no. 4, 613–621, 10.1002/jbm.10167, 2-s2.0-0037097175. 11948520

[31] Sill T. J. and von Recum H. A. , Electrospinning: Applications in Drug Delivery and Tissue Engineering, Biomaterials. (2008) 29, no. 13, 1989–2006, 10.1016/j.biomaterials.2008.01.011, 2-s2.0-40049090999. 18281090

[32] Bhardwaj N. and Kundu S. C. , Electrospinning: A Fascinating Fiber Fabrication Technique, Biotechnol Adv. (2010) 28, no. 3, 325–347, 10.1016/j.biotechadv.2010.01.004, 2-s2.0-77949652722. 20100560

[33] Beachley V. and Wen X. , Effect of Electrospinning Parameters on the Nanofiber Diameter and Length, Materials Science & Engineering, C: Materials for Biological Applications. (2009) 29, no. 3, 663–668, 10.1016/j.msec.2008.10.037, 2-s2.0-64749093059. 21461344 PMC3065832

[34] Homaeigohar S. and Boccaccini A. R. , Nature-Derived and Synthetic Additives to Poly(ɛ-Caprolactone) Nanofibrous Systems for Biomedicine; an Updated Overview, Frontiers in Chemistry. (2022) 9, 10.3389/fchem.2021.809676. PMC8807494 35127651

[35] Christen M. O. and Vercesi F. , Polycaprolactone: How a Well-Known and Futuristic Polymer has Become an Innovative Collagen-Stimulator in Esthetics, Clinical, Cosmetic and Investigational Dermatology. (2020) 13, 31–48, 10.2147/CCID.S229054. 32161484 PMC7065466

[36] Dwivedi R. , Kumar S. , Pandey R. et al., Polycaprolactone as Biomaterial for Bone Scaffolds: Review of Literature, Journal of Oral Biology and Craniofacial Research. (2020) 10, no. 1, 381–388, 10.1016/j.jobcr.2019.10.003. 31754598 PMC6854079

[37] Carriero V. C. , Di Muzio L. , Petralito S. , Casadei M. A. , and Paolicelli P. , Cryogel Scaffolds for Tissue-Engineering: Advances and Challenges for Effective Bone and Cartilage Regeneration, Gels. (2023) 9, no. 12, 10.3390/gels9120979. PMC10742915 38131965

[38] He Y. , Wang C. , Wang C. , Xiao Y. , and Lin W. , An Overview on Collagen and Gelatin-Based Cryogels: Fabrication, Classification, Properties and Biomedical Applications, Polymers. (2021) 13, no. 14, 10.3390/polym13142299. PMC8309424 34301056

[39] Mhatre A. , Bhagwat A. , Bangde P. , Jain R. , and Dandekar P. , Chitosan/gelatin/PVA Membranes for Mammalian Cell Culture, Carbohydrate Polymer Technologies and Applications. (2021) 2, 10.1016/j.carpta.2021.100163.

[40] Rodrigues S. , Dionísio M. , López C. R. , and Grenha A. , Biocompatibility of Chitosan Carriers With Application in Drug Delivery, Journal of Functional Biomaterials. (2012) 3, no. 3, 615–641, 10.3390/jfb3030615. 24955636 PMC4030999

[41] Jayakumar R. , Prabaharan M. , Nair S. V. , and Tamura H. , Novel Chitin and Chitosan Nanofibers in Biomedical Applications, Biotechnol Adv. (2010) 28, no. 1, 142–150, 10.1016/j.biotechadv.2009.11.001, 2-s2.0-71249135885. 19913083

[42] Jiménez-Gómez C. P. and Cecilia J. A. , Chitosan: A Natural Biopolymer With a Wide and Varied Range of Applications, Molecules. (2020) 25, no. 17, 10.3390/molecules25173981. PMC7504732 32882899

[43] Guillén-Carvajal K. , Valdez-Salas B. , Beltrán-Partida E. , Salomón-Carlos J. , and Cheng N. , Chitosan, Gelatin, and Collagen Hydrogels for Bone Regeneration, Polymers. (2023) 15, no. 13, 10.3390/polym15132762. PMC10346300 37447408

[44] Caliari S. R. and Harley B. A. C. , Collagen–Gag Materials, Comprehensive Biomaterials. (2011) 279–302, 10.1016/b978-0-08-055294-1.00075-1.

[45] Razavi M. , Qiao Y. , and Thakor A. S. , Three-Dimensional Cryogels for Biomedical Applications, Journal of Biomedical Materials Research, Part A. (2019) 107, no. 12, 2736–2755, 10.1002/jbm.a.36777, 2-s2.0-85071255538. 31408265 PMC7929089

[46] Rogers Z. J. and Bencherif S. A. , Cryogelation and Cryogels, Gels. (2019) 5, no. 4, 10.3390/gels5040046. PMC6956035 31816989

[47] Boakye M. A. D. , Rijal N. P. , Adhikari U. , and Bhattarai N. , Fabrication and Characterization of Electrospun PCL-MgO-Keratin-Based Composite Nanofibers for Biomedical Applications, Materials. (2015) 8, no. 7, 4080–4095, 10.3390/ma8074080, 2-s2.0-84937713936. 28793426 PMC5455672

[48] Qi H. and Coplen T. B. , Investigation of Preparation Techniques for *δ*2H Analysis of Keratin Materials and a Proposed Analytical Protocol, Rapid Communications in Mass Spectrometry. (2011) 25, no. 15, 2209–2222, 10.1002/rcm.5095, 2-s2.0-80052808361. 21735504

[49] Bajestani M. I. , Kader S. , Monavarian M. , Mousavi S. M. , Jabbari E. , and Jafari A. , Material Properties and Cell Compatibility of Poly(γ-Glutamic Acid)-Keratin Hydrogels, International Journal of Biological Macromolecules. (2020) 142, 790–802, 10.1016/j.ijbiomac.2019.10.020. 31622720

[50] Xue J. , Wu T. , and Xia Y. , Perspective: Aligned Arrays of Electrospun Nanofibers for Directing Cell Migration, APL Materials. (2018) 6, no. 12, 10.1063/1.5058083, 2-s2.0-85058440175. PMC7743993 33335802

[51] Olevsky L. M. , Jacques M. , and Hixon K. R. , Porevision: A Program for Enhancing Efficiency and Accuracy in SEM Pore Analyses, Biorxiv. (2024) https://www.biorxiv.org/content/10.1101/2024.09.19.613935v1. 10.3390/gels11020132 PMC11855315 39996675

[52] Chalise R. , Niroula A. , Shrestha P. , Paudel B. , Subedi D. , and Khanal R. , A low-Cost Goniometer for Contact Angle Measurements Using Drop Image Analysis: Development and Validation, AIP Advances. (2023) 13, no. 8, 10.1063/5.0164668.

[53] Xue W. , Champ S. , Huglin M. B. , and Jones T. G. J. , Rapid Swelling and Deswelling in Cryogels of Crosslinked Poly (n-Isopropylacrylamide-Co-Acrylic Acid), European Polymer Journal. (2004) 40, no. 3, 467–476, 10.1016/j.eurpolymj.2003.11.004, 2-s2.0-0842309673.

[54] Hixon K. R. , Lu T. , Mcbride-Gagyi S. H. , Janowiak B. E. , and Sell S. A. , A Comparison of Tissue Engineering Scaffolds Incorporated With Manuka Honey of Varying UMF, Biomed Research International. (2017) 2017, 1–12, 10.1155/2017/4843065, 2-s2.0-85018604192. PMC5343224 28326322

[55] Hixon K. R. , Melvin A. M. , Lin A. Y. , Hall A. F. , and Sell S. A. , Cryogel Scaffolds From Patient-Specific 3D-Printed Molds for Personalized Tissue-Engineered Bone Regeneration in Pediatric Cleft-Craniofacial Defects, Journal of Biomaterials Applications. (2017) 32, no. 5, 598–611, 10.1177/0885328217734824, 2-s2.0-85033456312. 28980856

[56] Suarez-Arnedo A. , Torres Figueroa F. , Clavijo C. , Arbeláez P. , Cruz J. C. , and Muñoz-Camargo C. , An Image J Plugin for the High Throughput Image Analysis of in Vitro Scratch Wound Healing Assays, Plos One. (2020) 15, no. 7, 10.1371/journal.pone.0232565. PMC7386569 32722676

