# Supplementary material for: Keratin Additive for Cellular Adhesion in Transcutaneous Prosthetics
Source: J Tissue Eng Regen Med. 2025 Dec 30;2025:4337554. doi: 10.1155/term/4337554 (PMC12750100; doi:10.1155/term/4337554)
Supplement: Supplementary file 1 — Supporting Information Additional supporting information can be found online in the Supporting Information section. [file TERM-2025-4337554-s001.docx]

**Supplemental Figures**


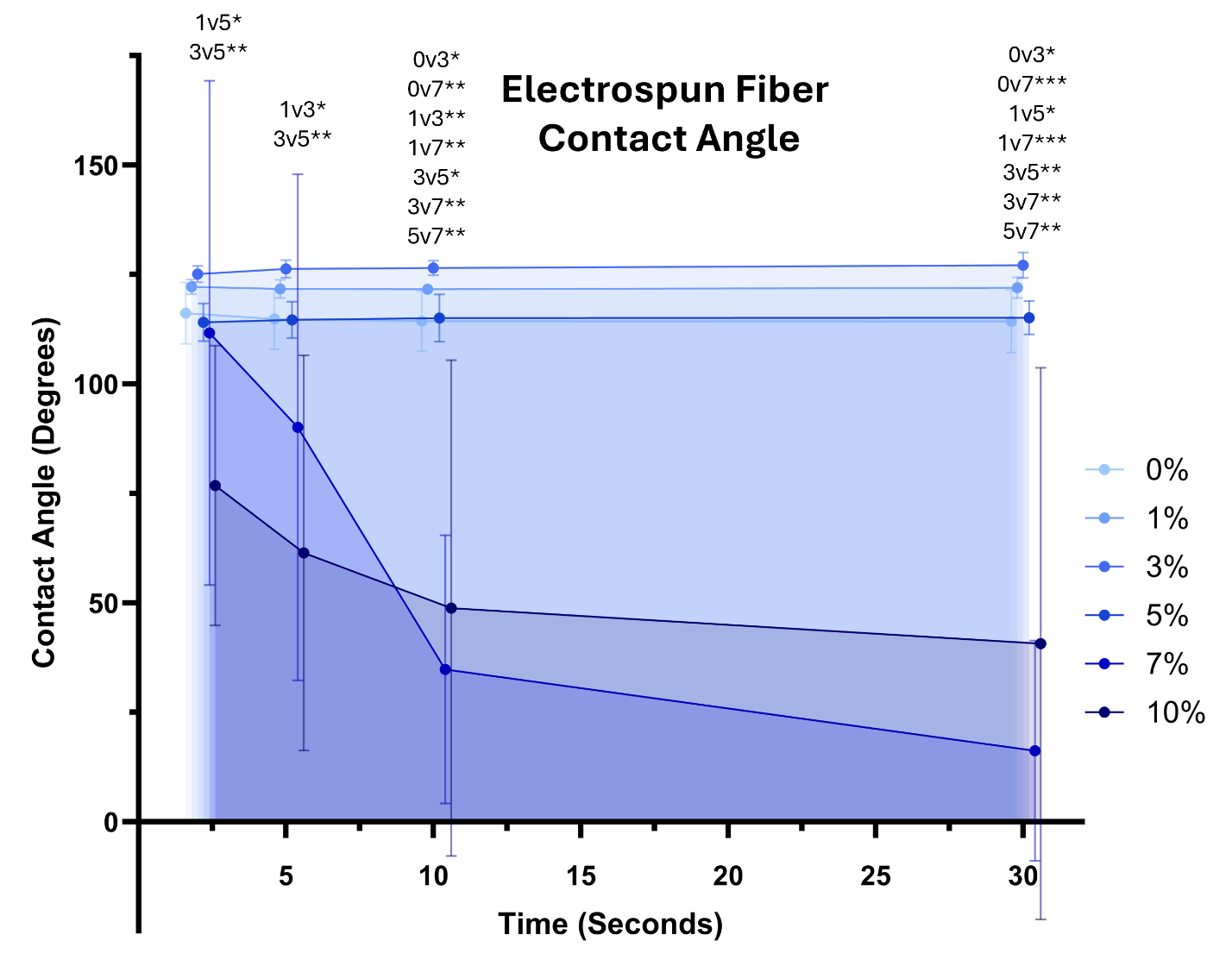


**Supplementary Figure 1:** Expanded contact angle analysis (N=3) showing a slight increase in contact angle with small keratin concentrations followed by a rapid and inconsistent drop in contact angle in the 7 and 10 wt/wt% groups. Detailed significance between the groups is from a 2-way ANOVA with Tukey correction for multiple comparisons with a single pooled variance test.


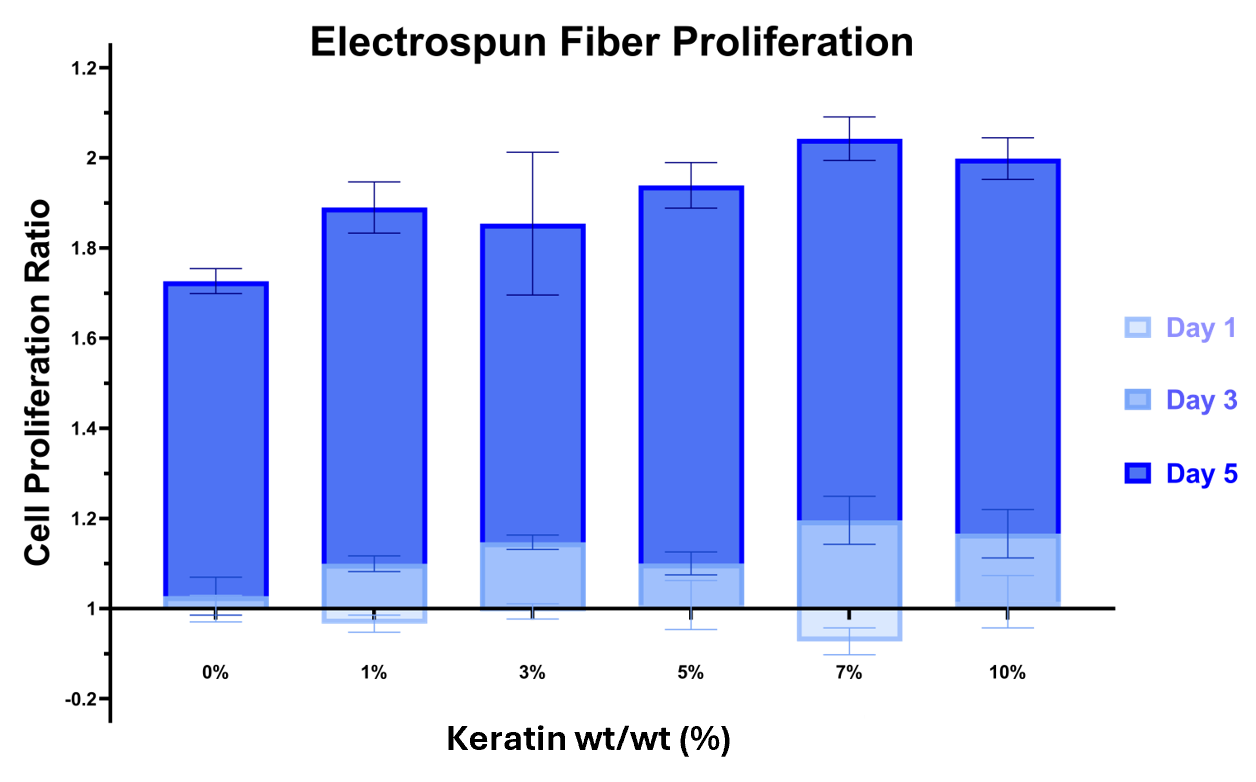


**Supplementary Figure 2:** Cell analysis showing scaffolds grouped by percentage with proliferation magnitude over days 1, 3, and 5 represented in a stacked bar. Cell proliferation analysis showing scaffolds grouped by day with significant differences only for the day 3 checkpoint between 0 and 3, 0 and 5, and 0 and 10 wt/wt%. Data was measured via 1-way ANOVA with Tukey's multiple comparisons test P<0.05.


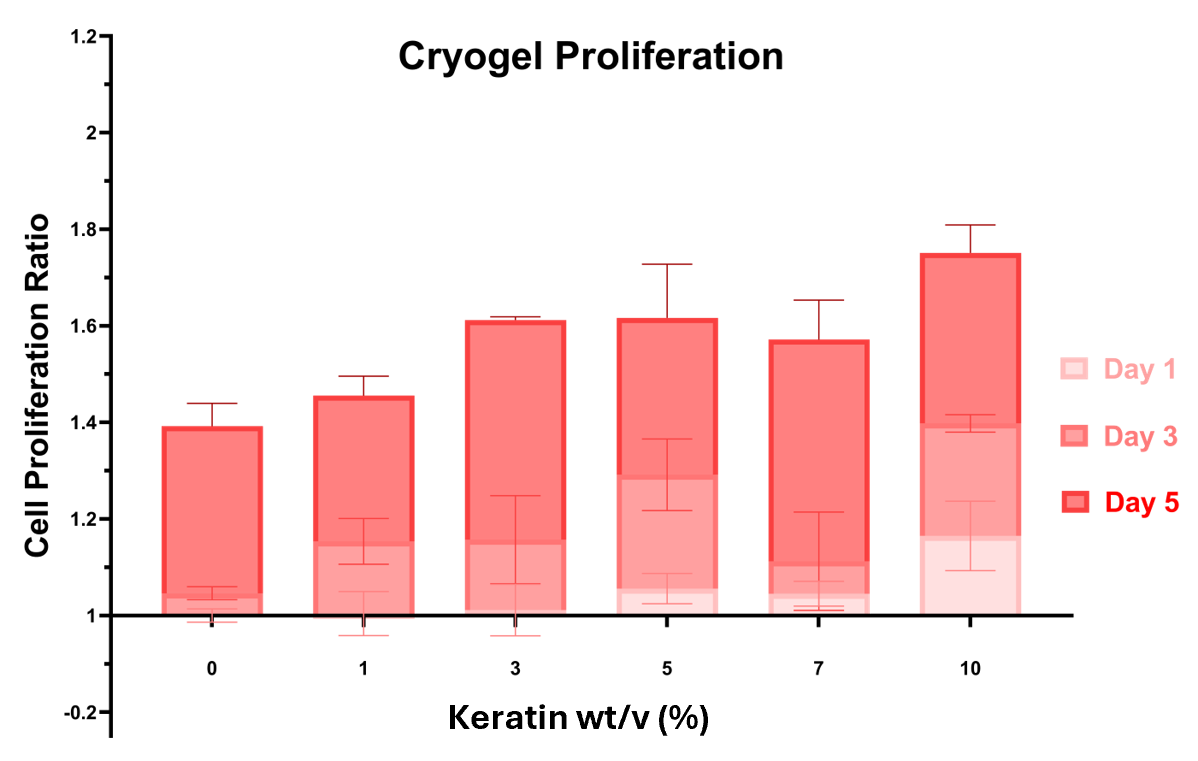


**Supplementary Figure 3:** Cell analysis showing scaffolds grouped by percentage with proliferation magnitude over days 1, 3, and 5 represented in a stacked bar. Day 1 indicates significant differences noted for 10% when compared with 0, 1, and 3 wt/v%. Day 3 showed significant differences when comparing 1 wt/v% to both 7 and 10 wt/v%. Day 5 then showed significant differences when comparing 1 wt/v% with 3, 5, and 10 wt/v% as well as when comparing 1 and 10 wt/v%. Significance was measured via 1-way ANOVA comparing within day checkpoint groups with Tukey's multiple comparisons test P<0.05.
